# Molecular dynamics simulations suggest possible activation and deactivation pathways in the hERG channel

**DOI:** 10.1038/s42003-022-03074-9

**Published:** 2022-02-24

**Authors:** Flavio Costa, Carlo Guardiani, Alberto Giacomello

**Affiliations:** Dipartimento di Ingegneria Meccanica e Aerospaziale, Sapienza Università di Roma, Via Eudossiana 18, 00184 Rome, Italy

**Keywords:** Computational biophysics, Permeation and transport

## Abstract

The elusive activation/deactivation mechanism of hERG is investigated, a voltage-gated potassium channel involved in severe inherited and drug-induced cardiac channelopathies, including the Long QT Syndrome. Firstly, the available structural data are integrated by providing a homology model for the closed state of the channel. Secondly, molecular dynamics combined with a network analysis revealed two distinct pathways coupling the voltage sensor domain with the pore domain. Interestingly, some LQTS-related mutations known to impair the activation/deactivation mechanism are distributed along the identified pathways, which thus suggests a microscopic interpretation of their role. Split channels simulations clarify a surprising feature of this channel, which is still able to gate when a cut is introduced between the voltage sensor domain and the neighboring helix S5. In summary, the presented results suggest possible activation/deactivation mechanisms of non-domain-swapped potassium channels that may aid in biomedical applications.

## Abstract (web summary)

Costa et al. present the electro-mechanical coupling between the voltage sensor and pore domain of the hERG channel using a combination of molecular dynamics simulations and theoretical network analyses.

## Introduction

The human ether-à-go-go-related gene K^+^ channel (hERG) is a voltage-activated cardiac^1^ channel involved in the repolarization phase of the action potential^2^. Pathological mutations of hERG, as well as promiscuous interactions with a wide range of chemicals^3,4^, can lead to premature or delayed repolarization of the cardiac membrane resulting in severe channelopathies referred to as short and long QT syndromes (SQTS and LQTS), respectively. Notwithstanding its biomedical importance, little is known on hERG mechanisms of activation and deactivation^5^, probably due to the fact that structural information of the channel is still incomplete, including the closed state. The present study aims at filling these gaps, providing a molecular-level understanding of the activation/deactivation mechanism(s) of hERG channel by molecular dynamics simulations.

Alterations in the expression, trafficking, and opening and closing of the hERG gate are linked to serious diseases, including LQTS, that may lead to sudden cardiac death. Currently, 200 LQTS-associated hERG mutations have been reported and LQTS affects an estimated 1 in 5000–10,000 people worldwide^3^. The disease can also be induced by the unspecific interaction of hERG with a wide range of drugs including anti-arrhythmics, antibiotics, and antihistamines^4,6,7^. The severity of these conditions is such that regulatory agencies prescribe the testing of all new drugs for hERG block. In this context, the FDA and a consortium of other regulatory agencies have promoted the Comprehensive in Vitro Proarrhythmia Assays^8,9^, a large-scale screening program to test candidate new drugs for cross-reactivity and block of hERG channel.

**Fig. 1:**
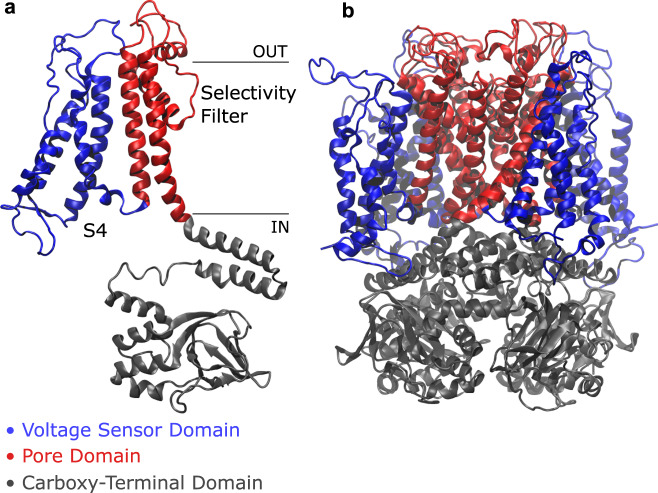
Side view of hERG channel. A single subunit (**a**) and the whole protein (**b**) are colored by domain: in blue, the voltage sensor domain ranging from helix S1 to loop L45, including the positively charged helix S4; in red, the pore domain, composed of helix S5, P-Loop, selectivity filter, and helix S6; in gray, the carboxy-terminal domain with the C-Linker and the cyclic nucleotide-binding homology domain.

Structurally, hERG is a homotetramer with each of the four identical subunits consisting of a voltage sensor domain (VSD, encompassing helices S1–S4) and a pore domain (PD, including helices S5 and S6) as well as N- and C-terminal cytosolic domains (CTD) with regulatory functions (Fig. 1).

During gating, helix S4, which carries five positively charged residues, senses the voltage change and moves up or down across the membrane transmitting via a yet elusive pathway the motion to the PD that, in turn, closes or opens the channel to ion passage. The aim of this work is to clarify the molecular mechanism that couples the motion of helix S4 and the opening or closing of the gate at the cytosolic end of helix S6.

The hERG channel normally cycles over three different states: closed, open, and inactivated. The channel is closed at negative voltages. Membrane depolarization slowly opens the channel but inactivation^10^ occurs so rapidly that very little K^+^ current can be observed in the early phase of the action potential. When the membrane starts repolarizing, hERG quickly re-opens recovering from inactivation and favoring the rapid repolarization that terminates the cardiac action potential^11^. A cryo-electron microscopy structure of the open state was obtained by Wang and MacKinnon^12^ at 3.8 Å resolution (PDB ID 5VA2). In this structure, the voltage sensor (helix S4) adopts a depolarized conformation and the pore is open. An experimental structure of the closed state of hERG is currently not available. However, Whicher and MacKinnon^13^ determined the single-particle cryo-EM structure of the mammalian EAG1 channel in complex with calmodulin. EAG1 is a member of the EAG family like hERG so that the two channels have high sequence identity and, overall, exhibit a similar architecture. This makes EAG1 a good template for the homology modeling of the closed state of hERG. Indeed the structure of EAG1 shows a closed pore but the S4 helix is in the open conformation. Experiments^14^ suggest that, in the closed state, each S4 helix should be displaced downwards by one or two residues; however, the exact position of S4 is unknown and will be investigated in the present work.

Voltage-gated potassium channels can be classified in 11 subfamilies^15^, the first 9 (Shaker-type) exhibiting a domain-swapped architecture and the remaining two (including hERG) a non-domain-swapped motif. Domain-swapped channels are characterized by such a long helical S4–S5 linker (also referred to as L45) that the VSD of a subunit interacts with the PD of the neighboring subunit rather than that of the same subunit; this long linker acts as a mechanical lever pushing onto helix S6 and straightening it to close the pore—these are the main features of the classical gating mechanism of domain-swapped channels^15^. In hERG and other non-domain-swapped channels, however, the S4–S5 linker is non-helical and so short that it is unlikely to exert a force on S6 (Fig. 1). Moreover, some split channels, i.e., constructs where the covalent continuity of the linker is interrupted, showed gating behavior comparable to that of the wild type^16,17^, thus suggesting that the S4–S5 linker in non-domain-swapped channels does not act as a simple force transducer. An alternative gating mechanism is therefore to be sought for.

Understanding the activation/deactivation mechanisms of hERG amounts to understanding how a voltage-induced conformational change of the sensor helix S4 propagates to the cytosolic end of the S6 helix bundle where the gate is located. Such a long-range conformational transition suggests that voltage-gated ion channels^18^, similarly to ligand-gated ones^19^, behave as allosteric molecules. In a typical allosteric enzyme, the binding of a ligand to the allosteric pocket triggers a conformational change that propagates across the protein structure, finally affecting the binding affinity of the orthosteric site. A commonly employed approach to study allosteric proteins examines how the fluctuations of residue pairs correlate over the course of a simulation to reconstruct the propagation of the motion across the protein network^20^. This approach was successfully employed to study the long-range communication path in the E2 enzymes^21^ and in the tRNA synthetases^22^. This approach has recently been applied also to ion channels^23,24^, in particular for the domain-swapped Kv1.2/2.1 chimera. In Ref. ^23^, two main communication pathways were identified, corresponding to the classical electro-mechanical lever mechanism and to a non-canonical communication pathway between helix S4 of subunit *n* and helix S5 of the neighboring subunit. In the latter case, the path moves up along S4, jumps onto the extracellular end of helix S5 of a neighboring subunit, goes down along S5, and finally reaches the C-terminal end of helix S6; the authors predicted this mechanism to be more important for non-domain-swapped channels. In this work this challenge was taken up, applying a network theoretical approach to the hereby unknown hERG activation/deactivation mechanisms.

In this work, in the first place, a putative structure of the closed conformation is provided, analyzing several conformations characterized by different gating charges. Secondly, the combination of molecular dynamics and network analysis allowed us to reveal two activation/deactivation mechanisms, one involving helices S4→L45→S5→S6 or S4→L45→S6 and the other one S4→S1→S5→S6, similar to the non-canonical path observed in domain-swapped channels^23,25^. A set of positive and negative control simulations of mutants further confirmed the validity of the predicted communication paths. In addition, split channels were simulated, providing mechanistic insights into the counterintuitive experimental finding that hERG activation/deactivation are impaired only when the cut is close to the S4 end^17^.

The paper is organized as follows. In the Results section, a homology model of the hERG closed state was built. The analysis of activation/deactivation pathways from the VSD to the PD is further reported, identifying two pathways and clarifying the role of loop L45 by simulations of split channels. Finally, it is shown that hERG activation/deactivation can be described in terms of a few geometric observables. In the Discussion section, the conclusions of this work are drawn, including its relevance for inherited and induced LQTS.

## Results

### Structure of the closed state

The open (O) state was produced from the experimentally solved structure (PDB ID 5VA2)^12^ and the missing loops were modeled with MODELLER^26^. In the structure of the open state, a very long fragment (residues 132-397) connecting the PAS domain with the VSD was not experimentally resolved. Since this fragment was too long to be reliably modeled using even state-of-the-art bioinformatic tools, the whole N-terminal region (residues 1-397) was not included. The structure studied in this work therefore approximately corresponds to the hERG1b isoform^27–29^ which lacks the entire PAS/Pas-cap domains (i.e., is 340 residues shorter than hERG1a). The hERG1b isoform was recently found to be critical for human cardiac repolarization as testified by a hERG1b-specific mutation associated with intrauterine fetal death^30,31^.

For the closed (C) state no experimental structure was available. A homology model was initially produced using the EAG1 closed channel (PDB ID 5K7L)^13^ as a template but the resulting system inherited from the template a VSD in the O configuration. More in detail, the O or C conformation of helix S4 depends on its relative position with respect to the critical phenylalanine (F463) on helix S2 that acts as the gating charge transfer center (GCTC). In the O conformation, only two of the five positively charged residues of helix S4 are below the critical F463 ring. It is not clear how many basic residues must be below the critical F463 in the C state. Using the limiting slope method, the gating charge of hERG channel was experimentally estimated to be 6.4e^14^. The fact that the technique relies on the ability to measure open probabilities close to zero, however, introduces a strong uncertainty on the experimental measurements that must be considered as a lower bound of the true value. Assuming a 20% underestimation, Zhang et al.^14^ suggested a corrected value of 8.0e. The variability of the experimental estimates of the gating charge suggests the opportunity to simulate molecular systems with different displacements of helix S4 (Supplementary Fig. 1). In particular, in the spirit of Ref. ^32^, each VSD in the C state was moved down via steered molecular dynamics (SMD) simulations^33^ producing three isoforms of the C state with Q_*g*_: 4*e* with the four S4 helices displaced downwards by a single basic residue, 8*e* displaced by two basic residues, and 6*e* with S4 helices of subunit I and III displaced by one residue and the remaining ones by two residues (Supplementary Fig. 2). During the SMD simulation helices S1, S2, and S3 were kept fixed because no experimental evidence is known of their motion during the dynamics of the VSD^34^.

The stability of the closed state model was assessed by analyzing the root mean square deviation (RMSD). After a modest increase following the SMD run, RMSD remained roughly constant during the 100 ns of the production run (Supplementary Fig. 3b–d). Then, we focused on interactions that are experimentally known to stabilize the closed state of the channel: D411-K538^35^, D456-K525^35^, E544-R665^36,37^. As shown in Supplementary Fig. 4 and in Supplementary Table 1, in each of the closed structures we considered, these residues are closer than 5 Å on average, thus confirming the presence of said interactions in our systems. A more detailed discussion of the salt bridges chosen for the validation of the closed states can be found in Supplementary Note 1.

### Contact analysis: activation/deactivation hypotheses

**Fig. 2:**
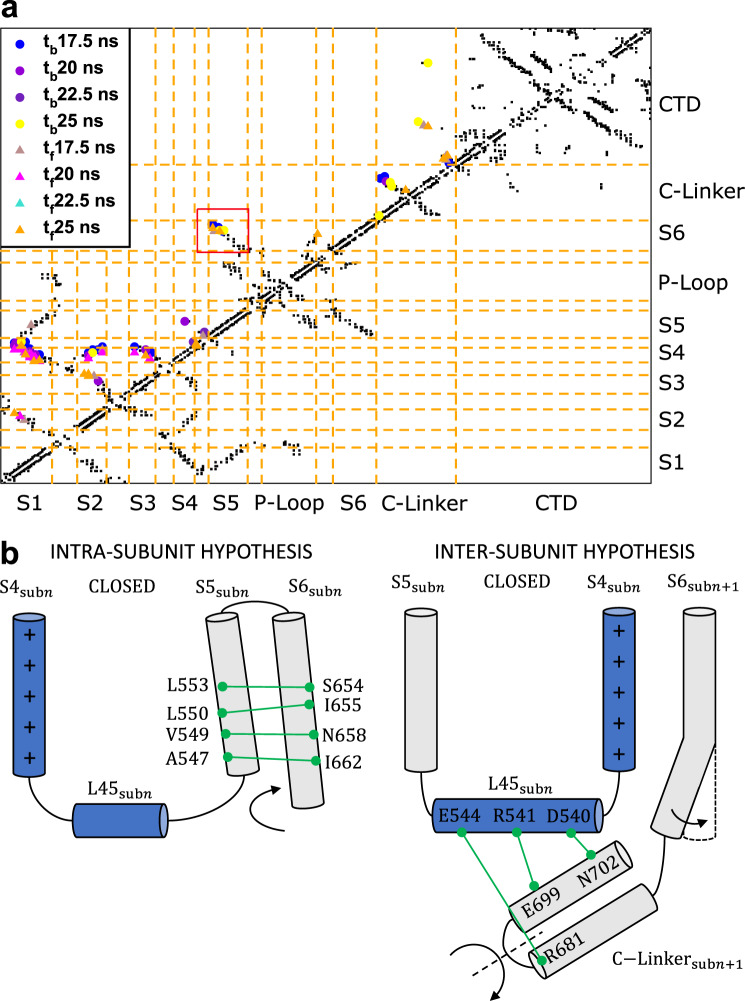
Contact analysis and activation/deactivation hypotheses. **a** Contact map of the first subunit of the system with Q_*g*_ = 8*e* during the transition from O to C state. The black dots represent the contacts in the initial O conformation not broken during the simulation while the colored symbols indicate the contacts formed or broken at different times (*t*_*f*_ and *t*_*b*_, respectively) during TMD. **b** Illustration of the two activation/deactivation hypotheses displaying the relevant conserved interactions in the C state.

The opening/closing of hERG was induced by targeted molecular dynamics (TMD) simulations^38^ using RMSD to drive the system from the O to the C state and vice versa. By computing a contact map for each frame of the TMD trajectories the sequence of contact breakdown and formation events could be followed (Fig. 2a). The identification of conserved, formed, or broken interactions in all subunits allowed us to formulate two hypothetical activation/deactivation mechanisms (Fig. 2b).

The first mechanism is an intra-subunit one based on the conserved interactions between helices S5 and S6 of the same subunit (red box in Fig. 2a): L553-S654, L550-I655, V549-N658, and A547-I662. The contact pattern shown also in Supplementary Figs. 5–7 suggests that helix S5 might directly push onto helix S6 of the same subunit causing its straightening and, consequently, the pore closure.

By analyzing the contact maps of the whole protein (Supplementary Fig. 8) conserved bonds between loop L45 of subunit *n* and the C-Linker of subunit *n* + 1 were noticed: D540-N702, R541-E699, and E544-R681. In this case, we hypothesized that the pressure exerted by loop L45 would induce a rotation of the C-Linker resulting in the straightening of helix S6 of subunit *n* + 1 and the pore closure. This mechanism is therefore inter-subunit. Contact maps of each subunit of the systems with gating charge 8*e*, 6*e*, and 4*e* are shown in Supplementary Figs. 5–7, respectively.

### Network analysis: activation/deactivation pathways

In order to discriminate the most realistic mechanism between those hypothesized from the contact analysis, the propagation of conformational changes inside the channel was studied employing a network approach, see^20,23^ for biophysics applications. In particular, the protein is represented as a graph where nodes correspond to protein residues and edges to interactions between pairs. Edges were assigned a weight based on the information distance ${d}_{{{ij}}}={{{{{{{\rm{-log}}}}}}}}\left|{{{{{{{{\rm{Corr}}}}}}}}}_{{{ij}}}\right|$, Corr_*ij*_ being the correlation coefficient (see Materials and Methods), that measured how efficiently information was transferred from one residue to the other. Using Dijkstra’s algorithm^39^, we computed the minimal paths between source and sink regions, which were chosen to be centered on helix S4 or loop L45 and on helix S6 (Supplementary Fig. 9), respectively. For each residue, a centrality index (CI) was computed as the fraction of minimal paths in which that residue was present.

**Fig. 3:**
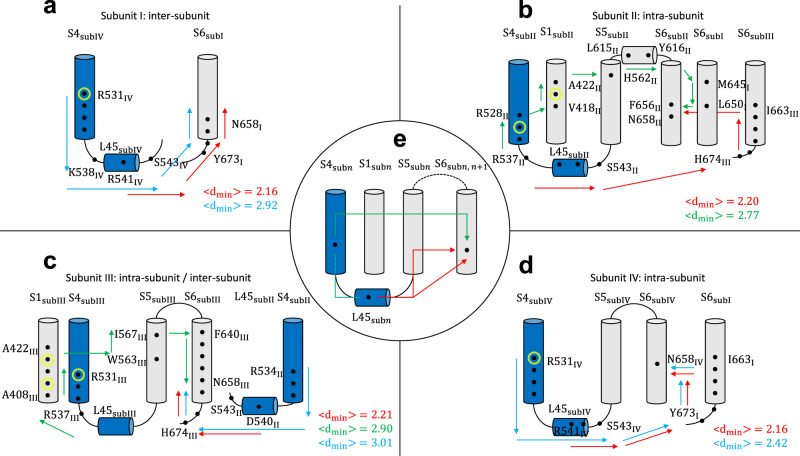
Activation/deactivation pathways of the closed system with Q_*g*_ = 8*e*. Paths identified in subunits I (**a**), II (**b**), III (**c**), and IV (**d**). Panel **e** schematizes the main families of activation/deactivation pathways identified in all systems. Arrows describe the preferred routes of motion propagation: blue arrows refer to S4→L45→S6 route; red arrows refer to the L45→S6 route; green arrows refer to S4→S1→S5→S6. Black dots correspond to residues on the path with CI > 0.15 (see Supplementary Table 1); yellow circles refer to the pathological mutation R531Q/W^42^ known to alter the gating of the channel inducing the LQTS. The mutations W410S, Y420C, and T421M impair both the trafficking and the gating^41^. Average minimal path lengths $\langle {d}_{\min }\rangle$ are also reported.

Two main families of paths that could be either intra- or inter-subunit (Fig. 3) were identified. In the first family, the motion was propagated downwards along helix S4 and then along loop L45. From there, the motion was propagated directly to helix S6 either belonging to the same or to the neighboring subunit (Fig. 3a–d); this mechanism resembles the intra-subunit activation/deactivation hypothesis in Fig. 2b.

In the second family of paths, the motion was propagated upwards along helix S4 before moving to S1 and S5 of the same subunit (Fig. 3b, c). From there, the path reached the helix S6 of the same subunit. This mechanism corresponds to the non-canonical path identified also in domain-swapped potassium channels^25^.

The two identified paths are not mutually exclusive but can coexist in the same channel molecule; they were identified also in the systems with gating charge 6*e* and 4*e* (Supplementary Figs. 10 and 11). Some interesting differences among the systems, however, were also detected. In the system with Q_*g*_ = 4*e*, there is an asymmetry between the paths originating from side S4 and side S5 of loop L45. In one of the four subunits, the paths starting from side S5 followed the L45→S5→S6 route while those starting from side S4 followed the L45→S4→S1→S5→S6 route. In the other three subunits, all the paths originating from loop L45 were qualitatively similar regardless of their starting point. In all of the four subunits, however, the paths originating from side S5 of L45 were significantly shorter than those starting from side S4, typically accounting for a more effective propagation of motion. This trend was also confirmed in the systems with gating charge 8*e* and 6*e*. These results show a functional asymmetry between the ends of loop L45 that is in agreement with experiments on hERG split channels^17^.

It is to remark that in the system with Q_*g*_ = 4*e*, the S4→L45→S5→S6 path was somewhat shorter than S4→S1→S5→S6 such that the latter is expected to be slightly less relevant in the dynamical coupling between S4–L45 and S6. In the system with Q_*g*_ = 6*e* the information transfer was generally less efficient than in the other systems. In particular, in the second and third subunits paths of average minimal length $\langle {d}_{\min }\rangle \approx 4$ in contrast with $\langle {d}_{\min }\rangle \approx 2$ in the other systems were found. Considering the logarithmic nature of this metric, a difference of two units in ${d}_{\min }$ corresponds to a difference of one order of magnitude in terms of correlations suggesting that in this system the S4–S6 coupling should be weaker. As a final remark, it was noted that none of the most efficient communication paths involved the C-Linker of the neighboring subunit disproving the hypothetical inter-subunit mechanism (Fig. 2b). A complete representation of the identified communication pathways is shown in Supplementary Fig. 12 together with the channel structures.

### Simulations of mutants

The network approach allowed us to identify two routes for motion propagation as the molecular basis of the VSD-PD coupling mechanism. It is known from the literature that the majority of the mutations that induce the LQTS impair the trafficking of hERG^40^. However, some mutations alter the gating mechanism of the channel. For instance, mutants T421M^41^ and R531Q^42^ are known to traffic normally while they are characterized by a depolarizing or hyperpolarizing shift of the half-maximal activation voltage *V*_1/2_, indicating an impairment of the activation or deactivation processes. These mutations lend themselves well to test the reliability of the results. If the VSD-PD coupling occurs through the predicted communication pathway, mutations known to affect activation/deactivation should be identified as pathway-breaking elements reducing the efficiency of motion propagation as measured by increasing path lengths. Therefore, we simulated mutants T421M and R531Q, which are experimentally known to impair gating in hERG. A more detailed description of the features of the two mutants can be found in the Methods section Choice of mutants. As shown in Supplementary Figs. 13 and 14, in both cases, activation/deactivation follow qualitatively similar routes as compared to the wild type but their length is significantly increased, underscoring that the VSD-PD coupling becomes weaker. As a negative control, we also simulated two random mutants not lying along the predicted communication pathways, A505V and F627A. As shown in Supplementary Figs. 15 and 16, these mutants do not affect either the communication pathways or their length that remain unchanged as compared to the wild type. The contact maps of all mutants are shown in Supplementary Fig. 17.

### Simulations of split channels

**Fig. 4:**
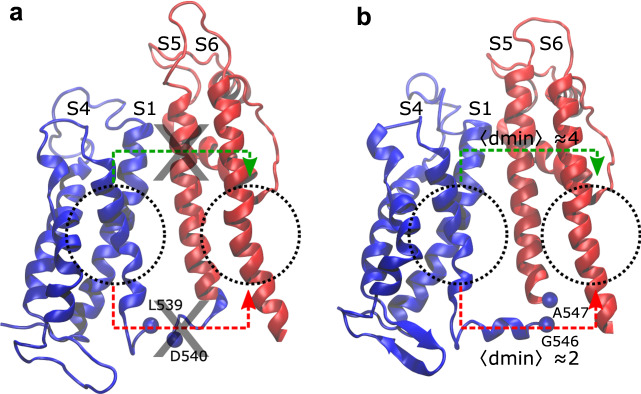
Communication pathways in split channels. Split channels disconnected before D540 (**a**) and after G546 (**b**): dotted circles are centered on the source and sink regions used in the network analysis; these locations correspond to helices S4 and S6; red arrows schematize the S4→L45→S6 and S4→L45→S5→S6 paths while green arrows refer to S4→S1→S5→S6 path. Average minimal path lengths $\langle {d}_{\min }\rangle$ are reported in the G546-split system (**b**); no pathways are found for D540 (**a**).

In order to further clarify the activation/deactivation mechanisms and confirm the role of the L45 loop, we reproduced in silico the split channels of Ref. ^17^, which show experimentally that cutting the L45 loop near the S4 helix causes a destabilization of the C state while channels split near the S5 helix open and close like the wild type. Starting from the pre-equilibrated C states, two split channels were produced introducing a disconnection before D540 and after G546, respectively (Fig. 4). In agreement with the literature^17^, no communication path was detected in channels split before D540 (Fig. 4a), while those cut after G546 exhibited the same activation/deactivation pathways as in the wild type (Fig. 4b). More specifically, in the G546-split system, the canonical path S4→L45→S6 followed the same route as in the wild type and had approximately the same length (ca. 2). Moreover, the non-canonical path S4→S1→S5→S6 was also qualitatively similar to that of the wild type, but somewhat longer, $\langle {d}_{\min }\rangle \approx$4.

In the D540-split channel, no path was identified. Unexpectedly, even the non-canonical activation/deactivation pathway, which does not pass through the L45 loop (green line in Fig. 3e), could not be found. This result could be justified observing that in the G546-split channel, the S4–S1 distance matched that of the C state in the wild type, whereas in the D540-split construct the S4–S1 distance was larger than that of the wild-type C state reaching values close to that of the O state at the bottom of the two helices (Supplementary Figs. 18 and 19). These results confirm the difficulty of the D540-split channel to reach the C state highlighted in experiments^17^ and rationalize the pattern in terms of an interruption of both communication pathways.

### Pseudo-potential of mean force (PMF)

**Fig. 5:**
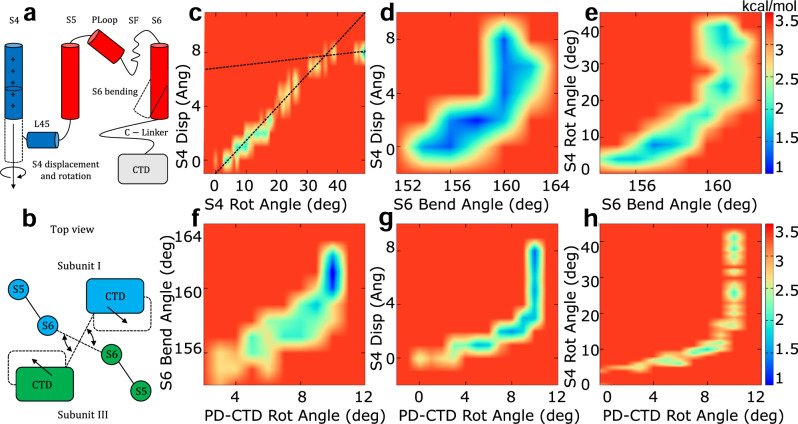
Pseudo-potential of mean force for hERG with Q_*g*_ = 8*e* as a function of geometrical descriptors, relevant for the O→C transition. The geometrical observables are: displacement and rotation of helices S4 and bending of helices S6 (**a**); rotation of the cytosolic domains with respect to the transmembrane ones (**b**). Pseudo-PMF maps as follows: S4 rotation vs S4 displacement (**c**); S6 bending vs S4 displacement (**d**); S6 bending vs S4 rotation (**e**); PD-CTD rotation vs S6 bending (**f**); PD-CTD rotation vs S4 displacement (**g**); PD-CTD rotation vs S4 rotation (**h**). Dashed lines are a guide to the eye. Bin widths are as follows: S4 displacement: 1.0 Å; S4 rotation: 2°; S6 bending: 1°; PD-CTD rot: 1°. Free energies were expressed in kcal/mol.

Useful mechanistic insights in the hERG activation/deactivation mechanisms are provided by a few geometrical observables that were monitored during the transition (Fig. 5a, b): displacement and rotation of helices S4; bending of helices S6; rotation of the CTD with respect to the transmembrane ones. Since the TMD simulations were run out of equilibrium, the calculation of a potential of mean force (PMF) is, strictly speaking, not possible. The driving speed is however sufficiently slow to allow to compute the pseudo-PMF graphs shown in Fig. 5, see Methods for details.

In Fig. 5c, the rotation and displacement of helix S4 proceed simultaneously almost throughout their entire range of values. However, when the roto-translation process is almost completed, the low free-energy region of the PMF shows a decrease in slope which means that rotation continues even after the translation has come to an end. Near the point of slope change a discontinuity indicates the presence of a PMF barrier, probably depending on steric clashes that slowed the rotation. In the systems with gating charge Q_*g*_ = 6*e* and Q_*g*_ = 4*e* the change in slope tends to occur earlier and the discontinuity is more pronounced (Supplementary Figs. 20a and 21a). Figure 5d, e shows that helix S6 bending and the roto-translation of helix S4 also proceed simultaneously for a large part of their range of values. However, after helix S6 bending has reached its maximum value, the roto-translation of helix S4 continues. This observation confirms our intra-subunit activation/deactivation hypothesis: since helix S4 is close to helix S5, a small initial motion of helix S4 is sufficient to induce a correspondingly small motion of helix S5 that pushes onto S6 inducing its bending. The function of helix S4, however, is not limited to the propagation of motion to helix S6. The ongoing motion of helix S4 after the bending of helix S6 is completed is probably finalized to the establishment of contacts (with a possible regulatory function) between L45 and the C-linker and/or the CTD. This pattern is also displayed by the systems with gating charge Q_*g*_ = 6*e* (Supplementary Fig. 20b, c) and Q_*g*_ = 4*e* (Supplementary Fig. 21b, c).

## Discussion

In this work, we have used molecular dynamics simulations combined with a network theoretical approach to reveal possible, hereby unknown mechanisms of hERG activation/deactivation. Whilst the proposed pathways will require experimental validation, our model makes a number of predictions that are consistent with previously published data. The first challenge in this investigation was the lack of an experimental structure of the C state. A homology model based on a template with high sequence identity (EAG1^13^) was built and the S4 helix from the experimental conformation was subsequently pulled down by SMD simulations. Using this approach, three systems were generated with gating charge Q_*g*_ = 4*e*, 6*e*, and 8*e* that differ in the number of S4 basic residues displaced below the GCTC. This choice was motivated by the uncertainty of the experimental value of the gating charge. Indeed, as already discussed, the value Q_*g*_ = 6.4*e* deriving from the limiting slope value is likely an underestimate of the true value and, even if an empirical correction yields a more likely value Q_*g*_ = 8*e*, we considered it more prudent to explore multiple gating scenarios. As a matter of fact, the analysis of activation/deactivation pathways revealed communication to be more efficient in the system with gating charge 8e than in the system with gating charge 6e. Our simulations thus are in agreement with the assumption that the limiting slope method underestimates the gating charge by 20%^14,43,44^. However, an ultimate answer on the conformation of the closed state of hERG will have to wait until an experimental structure (or structures) is resolved. In particular, a consensus has not been reached concerning even the number of open and closed states. Experimental evidence seems to suggest that a series of non-permeant structures exists, see, e.g., Liu et al.^45^ and Wang et al.^11^. These authors, performing tail current analysis, were able to study the time course of hERG activation excluding the complicating effect of fast inactivation. A substantial sigmoidal deviation from single-exponential activation was reported, which suggests the existence of multiple closed states. They also showed a voltage-independent step preceding activation which is a unique feature among potassium channels. Indeed, the study of deactivation tail currents also suggested that the open state may act as an aggregate conducting state, i.e., an ensemble of inter-converting conformations. Despite this evidence from electrophysiology and kinetic modeling studies^27^, the structural differences between these multiple open and closed states remain elusive, calling for more experimental and computational studies.

The analysis of pseudo-PMFs revealed that hERG dynamics can be described in terms of a few geometric variables. This approach, however, also raised a number of issues that will require extensive computational and experimental testing. A clue on the role of the rotation of CTD may be provided by the graph PD-CTD rotation vs S6 bending (Fig. 5f). The alignment of this graph along the diagonal suggests a possible auxiliary mechanism whereby the rotation of CTD is propagated to the C-Linker and from here to S6 helices. This trend, however, is only partially followed by the systems with gating charge 6*e* (first part of the trajectory; Supplementary Fig. 20d) and with gating charge 4*e* (last part of the trajectory; Supplementary Fig. 21d), so that more experimental validations are required. PMF results, for instance, pose the following question: what is the need for a long roto-translation of helix S4 if the extension of helix S6 is rapidly concluded? The contact analysis suggests that the motion of S4 is finalized to the establishment of contacts between loop L45 of subunit *n* and the C-Linker of subunit *n* + 1 (Fig. 2b). This leads to the tempting speculation of a coupling between the motion of helix S4 and the rotation of the CTD with respect to the transmembrane ones. This hypothesis is tested by analyzing the pseudo-PMFs PD-CTD rotation vs S4 displacement and PD-CTD rotation vs S4 rotation: Fig. 5g, h and Supplementary Figs. 20e, f and 21e, f clearly show that the rotation of the cytoplasmic domains ended before the motion of helix S4 was completed. As a result, the motion of helix S4 cannot be the cause of the rotation of the CTD, which still remains unclear. Concluding, the work excludes a causal role of S4 motion, so that the binding of the intrinsic ligand might play a role, possibly acting as a secondary voltage sensor^46^; at this stage, however, other factors cannot be ruled out.

Aside from the intriguing issue of the coupling between transmembrane domains and cytosolic domains, the activation/deactivation mechanisms of hERG, like that of all non-domain-swapped potassium channels, remained to date elusive. In domain-swapped channels, the long helical L45 linker acts as a mechanical lever pushing onto the neighboring S6 helix and thus closing the pore. In hERG, however, the L45 loop is too short to exert a force and the experiments on split channels show that mutants with an interrupted L45 have nearly normal activation/deactivation. Our contact analysis suggested two possible mechanisms: in the inter-subunit mechanism loop, L45 of subunit *n* pushes onto the C-Linker of subunit *n* + 1 causing a straightening of helix S6 and closure of the pore. Conversely, in the intra-subunit mechanism, loop L45 and helix S4 push onto helix S5 that transmits the pressure across its extensive interface with helix S6. The inter-subunit mechanism corresponds to the experimentally observed mode of action of KAT1 channel from Arabidopsis thaliana^47^. This kind of mechanism was also proposed for EAG1 channel^13^; the authors hypothesized that the downward displacement of helix S4 during hyperpolarization could play a role similar to the binding of calmodulin which was found to induce the rotation of the C-Linker. On the other hand, the hypothetical intra-subunit mechanism was supported by experiments on split channels^17^, showing that a covalent connection between VSD and PD is not necessary for the electro-mechanical coupling that can instead rely on the extensive network of anti-parallel non-covalent interactions between helices S5 and S6 and on the contacts established by the intracellular end of S4 and S5.

Network theoretical techniques allowed us to validate the hypothetical mechanisms by quantitatively assessing all possible communication pathways between helices S4 and S6. The analysis revealed two families of paths shown in Supplementary Movies 1–4 with the residues characterized by the highest value of the CI and the betweenness (Supplementary Table 2). The first essentially corresponds to the hypothetical intra-subunit mechanism and involves helices routes S4→L45→S5→S6 and S4→L45→S6. The second family, S4→S1→S5→S6, is similar to the non-canonical pathway identified in Ref. ^23^ in the study of the domain-swapped Kv1.2/2.1 chimera. The latter is also reminiscent of the non-canonical pathway recently discovered by Carvalho-de Souza and Bezanilla^25^ in the Shaker-like channel Kv1.2.

The analysis also highlighted an asymmetry between the routes originating from the opposite ends of loop L45 with the paths starting from the S5 side being significantly shorter. This asymmetry was confirmed by simulations of the C state of split channels with a cut in the S4–S5 linker. When the interruption was introduced after G546 (S5 end) pathways similar to those of the wild type were obtained. However, when the cut was placed before D540 (S4 end), no communication path could be found. Surprisingly, also the upper pathway was indirectly affected by the D540 cut because of the increased separation of helices S4–S1 and S1–S5. These results are consistent with experiments on split channels^17^, which showed an increasing difficulty to reach the C state as the split point was moved closer to the S4 end. Based on the simulations, these results can be interpreted as a greater impairment of both communication pathways when the cut point is close to the S4 end.

LQTS mutations are classified in four groups according to their mechanism of action: (1) abnormal transcription or translation; (2) deficient protein trafficking; (3) abnormal channel gating/kinetics; (4) altered channel permeability^40^. Experiments using mammalian expression systems show that up to 90% of LQTS missense mutations impair trafficking and thus belong to class (2). Recent studies, however, also identified a small but non-negligible group of mutations that affect gating. This is exactly the group of mutations whose molecular mechanism could be investigated with our network theoretical approach. A large-scale mutational analysis by Anderson et al.^40^ showed that mutations of class (3) can be found in the N- and C-terminal domains as well as in helix S6 of the pore. Mutations impairing the activation/deactivation process have also been mapped to helix S1^41^ and helix S4^42^. It is noteworthy that some of these class (3) mutations sit on the two activation/deactivation pathways identified with our network approach (yellow circles in Fig. 3 and in Supplementary Figs. 10 and 11). A set of positive and negative control simulations revealed that only mutations of residues sitting on the communication pathways impair the efficiency of information transfer lending further support to our model.

In short sum, our work provided a putative structure of the closed state of hERG channel and applied an approach combining molecular dynamics simulations and network theoretical techniques to explore possible communication pathways explaining the electro-mechanical coupling between VSD and PD. This approach, previously only applied to domain-swapped Shaker-like channels, may be extended to the study of other voltage-gated and ligand-gated ion channels leveraging their recently discovered nature of allosteric machines.

## Methods

### Production of the open and the closed states

hERG open state was produced from the experimentally solved structure (PDB ID 5VA2)^12^ and the missing loops were modeled using MODELLER^26^. For the C state, a homology model was initially produced using as a template the EAG1 closed channel (PDB ID 5K7L)^13^ featuring ca. 75% sequence identity. In order to pull the VSD to the close configuration, SMD simulations^33^ were run in the NVT ensemble for 2 ns applying a helix-axis-orthogonal force on the center of mass of C_*α*_ atoms of each S4 to move it toward S6 of the same subunit to prevent steric clashes; then a force parallel to the axis was applied for 4 ns to the center of mass of helix S4 to move it down. The pulling velocity was 3 Å/ns and the spring constant of the virtual spring between the dummy atom and the SMD atom was 10 kcal/mol/Å^2^. S1, S2, S3, and S5 were fixed and a helical restraint on S4 was applied with a force constant of 1000 kcal/mol/Å^2^. Using the CHARMM membrane builder^48,49^, all systems were embedded in bilayers of 1010 1-palmitoyl-2-oleoyl-sn-glycero-3-phosphocholine lipids with 49,467 TIP3P water molecules and 0.15 M of KCl to form a simulation box of ca. 125 × 125 × 150 Å totaling 223,584 atoms. The WHAT-IF server^50^ was used to analyze the protonation states of residues. All aspartates and glutamates were ionized. H5, H342, H374, H434 and H437 were predicted to be in the *δ* protonation state; H88, H95, H165, H181, H190, H277, H290, H306, H334, H365 and H454 were assigned to the *ϵ* protonation state.

### Mutants and split channels

Mutants were produced from the pre-equilibrated closed wild-type systems. At first, they were equilibrated for 6.5 ns in the NPT ensemble applying a time-varying harmonic restraint on each mutated residue and those closer than 5.0 Å. The force constant, initially set to 10 kcal/mol/Å^2^, was decreased by 2 units every 0.5 ns of this simulation and then they were equilibrated for 50 ns in the NPT ensemble.

The split channels were produced from the pre-equilibrated C states cutting the proteins between L539-D540 and G546-A547 of each subunit. They were equilibrated for 50 ns in the NPT ensemble.

### MD simulations

The equilibration run was carried out in the NPT ensemble for 100 ns. A first guess of the transition path from the O to the C state was obtained via RMSD-biased TMD^38^ simulations run for 25 ns in the NPT ensemble using a force constant of 100,000 kcal/mol/Å^2^ on C_*α*_ atoms. All simulations were run with NAMD^51^ using the ff14SB force field for the protein^52^ and the Lipid17 force field for the lipids^53^. The pressure was kept at 1.01325 Bar by the Nosé-Hoover Langevin piston method^54,55^ and the temperature was maintained at 303.15 K by a Langevin thermostat with a damping coefficient of 1 ps^−1^. Long-range electrostatic interactions were evaluated with the smooth Particle Mesh Ewald algorithm with a grid space of 1 Å. For short-range non-bonded interactions, a cutoff of 12 Å with a switching function at 10.0 Å was used. The integration time step was 2 fs.

### Contact analysis

In the WT the contact map was computed for each frame of the TMD simulation, considering two residues to be in contact when a pair of heavy atoms of the side-chains was closer than 5.0 Å. In mutants and split channels the analysis was carried out computing for each couple of residues the probability to be in contact during the equilibrium simulations. Precisely, two residues were considered to be in contact when a pair of heavy atoms of the side-chains was closer than 5.0 Å for at least 75% of the trajectory.

### Two-dimensional pseudo-PMF

Two-dimensional pseudo-PMFs were computed as a function of the geometric observables monitored during the transition: sliding and rotation of the S4 helices; bending of the S6 helices; rotation of CTD with respect to transmembrane ones (PD).

The bending angle *θ* of each helix S6 was computed as follows:

1 $$\theta = \arccos{\frac{{\overrightarrow{v_{CT}}}{\overrightarrow{v_{CB}}}}{|{\overrightarrow{v_{CT}}}||{\overrightarrow{v_{CB}}}|}}$$

where ${\overrightarrow{v_{CT}}}$ is a vector pointing from the middle region of helix S6 (residues 646-650) to the top of the same helix (residues 635-639). Similarly, ${\overrightarrow{v_{CB}}}$ is a vector pointing from the middle to the bottom end (residues 663-667) of helix S6.

The visual inspection of the open structure of hERG channel reveals that the transmembrane domains (VSD+PD) of non-neighboring subunits (1–3 and 2–4) are roughly orthogonal to the corresponding CTD. During the simulations, the rotation angles, *ϕ*_13_ and *ϕ*_24_, depart from this value and evolve as a function of time. They are defined by the following vectors: $\overrightarrow{{v}_{TMD}^{ij}}$: vector connecting the center of mass of the transmembrane domains (PD+VSD) of chain *i* with the center of mass of the transmembrane domains of non-neighboring chain *j*. $\overrightarrow{{v}_{CD}^{ij}}$: vector connecting the center of mass of the CTD (C-Linker + CTD) of chain *i* with the center of mass of the CTD of non-neighboring subunit *j*. Angles *ϕ*_13_ and *ϕ*_24_ are then computed as follows:

2 $${\phi_{13} = \arccos{\frac{\overrightarrow{{{{{{\mathrm{v}}}}}}^{13}_{{{{{\mathrm{TMD}}}}}}}\overrightarrow{{{{{{\mathrm{v}}}}}}^{13}_{{{{{\mathrm{CD}}}}}}}}{|\overrightarrow{{{{{{\mathrm{v}}}}}}^{13}_{{{{{\mathrm{TMD}}}}}}}||\overrightarrow{{{{{\mathrm{v}}}}}}^{13}_{{{{{\mathrm{CD}}}}}}|}}}$$

3 $${\phi_{24} = \arccos{\frac{\overrightarrow{{{{{{\mathrm{v}}}}}}^{24}_{{{{{\mathrm{TMD}}}}}}}\overrightarrow{{{{{{\mathrm{v}}}}}}^{24}_{{{{{\mathrm{CD}}}}}}}}{|\overrightarrow{{{{{{\mathrm{v}}}}}}^{24}_{{{{{\mathrm{TMD}}}}}}}||\overrightarrow{{{{{{\mathrm{v}}}}}}^{24}_{{{{{\mathrm{CD}}}}}}}|}}}$$

The rotation of helix S4 around its axis was computed as follows. In a perfect helical geometry, the vector connecting the center of mass of C_*α*_ atoms *n* and *n* + 4 with the C_*α*_ atom *n* + 2 is orthogonal to the helix axis. The angle *α* between this vector $\overrightarrow{{v}^{rot}}(t)$ and its counterpart $\overrightarrow{{v}^{rot}}(0)$ in the initial conformation defines the rotation angle. To account for local deformations of the helical geometry that might undermine the orthogonality of $\overrightarrow{{v}^{rot}}$ to the axis, the calculation was performed using the component of $\overrightarrow{{v}^{rot}}$ orthogonal to the axis:

4 $${\alpha = \arccos{\frac{\overrightarrow{{{{{{\mathrm{v}}}}}}^{{{{{\mathrm{rot}}}}}}_{\bot}}({{{{{\mathrm{t}}}}}})\overrightarrow{{{{{{\mathrm{v}}}}}}^{{{{{{\mathrm{ro}}}}}}t}_{\bot}}(0)}{|\overrightarrow{{{{{{\mathrm{v}}}}}}^{{{{{{\mathrm{rot}}}}}}}_{\bot}}({{{{{\mathrm{t}}}}}})||\overrightarrow{{{{{{\mathrm{v}}}}}}^{{{{{{\mathrm{rot}}}}}}}_{\bot}}(0)|}}}$$

Defining $\overrightarrow{{r}_{COM}}(t)$ as the vector corresponding to the center of mass of helix S4, the displacement of S4 along its axis is:

5 $${{{{{\mathrm{d}}}}}} = [{\overrightarrow {{{{{{\mathrm{r}}}}}}_{{{{{{\mathrm{COM}}}}}}}}}({{{{{\mathrm{t}}}}}}) - {{\overrightarrow {{\mathrm {r}}_{{{{{{\mathrm{COM}}}}}}}}}}(0)]\hat{{{{{\mathrm{h}}}}}}$$

where $\hat{h}$ is the versor aligned along S4 axis.

These quantities were used to calculate two-dimensional pseudo-PMFs:

6 $${{{{{{{\rm{PMF}}}}}}}}({{z}},{{x}})=-{{{k}}}_{{{B}}}{{T}}\log {{H}}({{x}},{{y}})$$

where *k*_*B*_ is the Boltzmann constant, *T* is the temperature set at 300.0 K and *H*(*x*, *y*) is a normalized two-dimensional histogram of the two observables *x* and *y*. The histograms and pseudo-PMFs calculated for each system were: S4 rotation angle vs S4 displacement; S6 bending angle vs S4 displacement; S6 bending angle vs S4 rotation angle; PD-CTD rotation angle vs S4 displacement; PD-CTD rotation angle vs S4 rotation angle; PD-CTD rotation angle vs S6 bending angle. The displacement and rotation angle of the S4 helices and the bending angle of the S6 helices were the averages over the four subunits. For the rotation angle of S4 helices, the average of the rotation angles over the four subunits were computed in the middle and at the bottom of individual S4 helices. Finally, the PD-CTD rotation angle was computed as the average of *ϕ*_13_(*t*) and *ϕ*_24_(*t*).

### Network analysis

The pathway communication analysis was carried out representing the protein as a graph^56^ where nodes are protein residues and edges interactions between pairs. Edge weights were calculated using: ${d}_{{{ij}}}=\,-\log \left|{{{{{{{{\rm{Corr}}}}}}}}}_{{{ij}}}\right|$, where Corr_*ij*_ is the correlation coefficient as the normalized covariance of C_*α*_ positions:

7 $${{{{{{{{\rm{Corr}}}}}}}}}_{{{ij}}}=\frac{\langle ({\overrightarrow{r}}_{i}-\langle {\overrightarrow{r}}_{i}\rangle )({\overrightarrow{r}}_{j}-\langle {\overrightarrow{r}}_{j}\rangle )\rangle }{\sqrt{\langle {({\overrightarrow{r}}_{i}-\langle {\overrightarrow{r}}_{i}\rangle )}^{2}\rangle \langle {({\overrightarrow{r}}_{j}-\langle {\overrightarrow{r}}_{j}\rangle )}^{2}\rangle }}$$

where ${\overrightarrow{r}}_{i}$ and ${\overrightarrow{r}}_{j}$ are the position vectors of the *i* and *j* residues and −1 < Corr_*ij*_ < 1. In building the graph two residues are considered linked by an edge only if their communication distance *G* is below a cutoff *G*_cut_ = 0.40. This means that only pairs of residues with an absolute value of the correlation coefficient greater than 0.67 are connected by an arc. Supplementary Note 2 outlines how the properties of the network and the communication pathways are affected by *G*_cut_ and justifies the value of this cutoff chosen in our work. Spheres of radius 7 Å were centered on two key residues on helix S4 or loop S4–S5 and on S6 of the same or *n* + 1 subunit to identify all residues inside them for at least 70% of the trajectory defining the source and sink regions (Supplementary Fig. 9). The shortest pathways were computed using Dijkstra’s algorithm^39^. The betweenness of each residue was computed with Brandes algorithm^57^ as implemented in the NetworkX library^58^. Our protocol of pathway calculation is clarified through a step-by-step example illustrated in Supplementary Note 3, in Supplementary Figs. 22–25 and Supplementary Table 3.

### Choice of mutants

The mutations T421M^41^ and R531Q^42^, lying at different levels along our communication pathways were simulated and analyzed with the network theoretical approach to assess their impact on the efficiency of communication transfer. We chose these two mutants because their careful experimental characterization has highlighted an influence on activation/deactivation rather than on trafficking.

During the course of its maturation^40,41^, the hERG channel undergoes the first glycosylation at N598 in the endoplasmic reticulum to generate a 135-kDa immature glycoprotein. The second and final glycosylation occurs in the Golgi to produce the mature 155-kDa form. This process suggests a simple experimental test to identify the trafficking deficient mutants. Indeed, in trafficking-competent mutants, as well as in the wild type, the western blot analysis resolves two distinct bands corresponding to the 135-kDa precursor and to the 155-kDa mature species. Conversely, in mutants with impaired trafficking, the 155-kDa band cannot be observed. Based on the western blot test, all the mutants analyzed in our study appeared to traffick normally^41,42^.

Moreover, performing whole-cell patch-clamp experiments, it is possible to record a current–voltage curve that can be fitted with a Boltzmann function^42^:

8 $$I=\frac{{I}_{{{{{{\rm{min}}}}}}}-{I}_{{{{{{\rm{max}}}}}}}}{(1+{e}^{(V-{V}_{1/2})/k})+{I}_{{{{{{\rm{max}}}}}}}}$$

where *I*_min_ is the minimally activated current, *I*_max_ is the maximally activated current, *V*_1/2_ is the voltage where 50% of the channels are open and *k* is a slope factor. When this analysis was run on R531Q^42^, *V*_1/2_ appeared to be shifted to depolarized voltages (from –14 to +32 mV) indicating an impairment of the C→O transition. A large depolarizing shift in *V*_1/2_ is also associated with mutations of residue T421. When Phan et al.^41^ performed alanine scanning of the residues of helix S1, the largest depolarizing shift was observed for the T421A mutation that exhibited a *V*_1/2_ of 16.1 mV. The LQTS mutation T421M was also characterized by a shift of *V*_1/2_ toward more positive potentials corresponding to an increase of the free energy of activation (with respect to the wild type) ΔΔ*G*_*act*_ = ~3.0 kcal/mol.

A more stringent validation of the model also requires negative control experiments. Within the framework of our work, this means simulating mutations not lying on the predicted communication pathways. Indeed, if the VSD-PD coupling is only realized through those pathways, mutations outside these routes should not directly affect the efficiency of information transfer and thus the length of the communication paths. Therefore, we chose to simulate mutants A505V and F627A, the former lying on helix S3 and the latter sitting on the selectivity filter. Moreover, according to Anderson et al.^40^ a slightly different mutation of residue F627 (F627L) at low temperatures exhibit normal activation/deactivation.

### Statistics and reproducibility

A block analysis was applied^59,60^ to characterize the variability of the information distances *G*_*ij*_ important for the reliability of the results. If each simulation is split in a number *N*_*B*_ of sufficiently long blocks, the matrices ${\overline{G}}_{B}$ computed in the various blocks can be considered as uncorrelated measurements. It is then possible to compute the variance of the mean for each element of the matrices:

9 $${\sigma }^{2}({({{\overline{G}}}_{ij})}_{B})=\frac{1}{{N}_{B}({N}_{B}-1)}\mathop{\sum }\limits_{i=1}^{{N}_{B}}{({{{({\overline{G}}_{ij}})}}_{B}-\,{ < }\,{{({\overline{G}}_{ij})}}_{B} \,{ > }\,)}^{2}$$

Specifically, the 100 ns (1000 frames) equilibrium trajectories of the closed systems were split into four blocks each of 25–ns (250 frames). Supplementary Table 4 shows the minimum, maximum and average value of the variance both considering all elements of the matrix or just the most correlated pairs of residues ($< {({{\overline{G}}}_{ij})}_{B} > \; < {G}_{{{{{{\rm{cut}}}}}}}$). In either case, the average variance is very small even if the range of variation is very large. Therefore, the variability along the trajectory of the information distance was investigated. To this end for each system (gating charge 4e, 6e, 8e) two representative pathways were chosen, a canonical one (S4→L45→S6) and a non-canonical one (S4→S1→S5→S6). If the pathway is represented as Res1→Res2→...→ResN, for each subsequent pair of residues along the path Res1→Res2, Res2→Res3, ... Res(*N*–1)→Res(*N*), the average information distance and the variance of the mean were considered. The variance analysis of the pathways for the systems with gating charge 4e, 6e, and 8e is reported in Supplementary Tables 5–7, respectively, showing that the variance of *G*_*ij*_ along the pathways is typically one to four orders of magnitude smaller than the average variance in the whole network. This pattern that strongly supports the reproducibility of the results, is probably due to an evolutionary pressure stabilizing the information distance along a sequence of residues important for the functional dynamics of the ion channel.

### Reporting summary

Further information on research design is available in the Nature Research Reporting Summary linked to this article.

## Supplementary information

Supplementary Information

Description of Additional Supplementary Files

Supplementary Movie 1

Supplementary Movie 2

Supplementary Movie 3

Supplementary Movie 4

Reporting Summary

## Data Availability

The PDB files of the closed states, the equilibration and the TMD trajectories that support the findings of this study are available in Zenodo with the identifier [10.5281/zenodo.5793755].

## Code availability

All software used for this study are available and the links are provided as follows. For the data collection: NAMD, CHARMM and MODELLER. For the data analysis: VMD and NetworkX (https://networkx.org/).
