# Supplementary material for: Molecular dynamics simulations suggest possible activation and deactivation pathways in the hERG channel
Source: Commun Biol. 2022 Feb 24;5:165. doi: 10.1038/s42003-022-03074-9 (PMC8873449; doi:10.1038/s42003-022-03074-9)
Supplement: Supplementary file 1 — Supplementary Information [file 42003_2022_3074_MOESM1_ESM.pdf]

Molecular Dynamics simulations suggest possible activation and deactivation pathways in the hERG channel

Flavio Costa[1], Carlo Guardiani[1], and Alberto Giacomello[1,*]

[1]Dipartimento di Ingegneria Meccanica e Aerospaziale, Sapienza Università di Roma, Via Eudossiana 18 00184 Rome, Italy
[*]Corresponding author: Alberto Giacomello, alberto.giacomello@uniroma1.it

Supplementary Notes

Supplementary Note 1: "Closed structures validation"

We generated the closed states of hERG channel through homology modelling and Steered Molecular Dynamics simulation. The structures can be assessed exploiting the rich body of experimental data in the literature. A first piece of experimental evidence pertains to the state-dependent patterns of salt bridges between the positively charged residues of helix S4 and the negatively charged residues on the other helices of the VSD. The different patterns of charge pairings enable the discrimination of closed and open states. Indeed, these studies date back to the period when no experimental structure of hERG was available and specific salt bridges provided spatial constraints for homology modelling. In particular, charge reversal mutagenesis experiments by Zhang et al [1] revealed that the K525D and K538D mutations significantly accelerated hERG activation suggesting these lysines could be involved in salt bridges stabilizing the closed state. This hypothesis was confirmed by a mutant cycle analysis showing that D411 (which is peculiar of the EAG family) at the inner end of S1 interacts with K538 at the inner end of S4, while D456 (conserved among Kv channels) at the outer end of S2 interacts with K525 at the outer end of S4. Interactions between charged residues in the trans-membrane segments of hERG may be important for the known slow activation rate of this channel. In fact, it can be speculated that the strong salt bridges stabilizing the closed state might be the cause of the slow movement of S4 [2] during membrane depolarization. As shown in Supplementary Figure 4, in all of the hERG closed states we generated, using a distance cutoff of 5.0 Å between the ϵ-Nitrogen of the lysines and the δ-Oxygen of the aspartates, we could identify both salt bridge D456-K525 and D411-K538.

Another group of contacts stabilizing hERG closed states are those between the S4-S5 loop and the C-terminal end of helix S6. In particular, Sanguinetti and coworkers [3, 4] showed that the D540K mutation endowed the hERG channel with the ability to open in response to membrane hyperpolarization. This unusual behaviour was shown to stem from the breakdown of the D540-R665 salt bridge and the electrostatic repulsion between the lysine placed in position 540 and R665. Another critical residue located on the S4-S5 loop is E544. While the mutation to lysine of this residue does not cause hyperpolarization-induced channel re-opening [3], the E544L mutation significantly enhances the pharmacologic response to hERG activator NS1643 (see [5] and references therein). Based on this evidence, Guo et al designed a set of powerful hERG activators with binding pocket in the neighborhood of D544. The efficacy of these drugs suggests that they prevent the formation of a critical salt bridge between E544 and a basic residue on helix S6. This mechanism is also supported by the observation [6] that covalently binding, through a disulfide bridge, a peptide mimicking the S4-S5 linker to the channel S6 C-terminus completely inhibits hERG. It is notable that in these experiments the disulfide bond was introduced between E544C and L666C, suggesting that in physiologic conditions a salt bridge is established between E544 and R665. Indeed, as illustrated in Supplementary Figure 4, salt bridge E544-R665 could be observed in all of our hERG closed states.

Supplementary Note 2: "Role of the G_{cut} parameter in the network analysis"

In the network analysis the critical parameter is represented by G_{cut}, the communication distance cutoff below which two nodes of the protein graph are connected by an arc. It differs from the physical distance between two residues (used in the contact map analysis) that comes into play only in a second stage, when the communication pathways computed through the network approach must be validated. Indeed, it may happen that two residues lie next to each other on a path (because their motion is highly correlated) but they are physically very far from each other, so that motion cannot be propagated from a residue to the other. The distance between two residues therefore determines whether a path is accepted or discarded but has no influence on the length and route followed by the pathway. These features instead depend on the G_{cut} parameter that is related to the minimal correlation coefficient required for two residues to be considered linked by an edge in the protein graph: $G_{cut} = -log|C|_{cut}$. In the network analysis we used G_{cut}=0.40 that corresponds to a minimum correlation $|C|_{cut}$=0.67. In order to show the role of the G_{cut} parameter, we repeated the pathway analysis with two values higher than 0.40 and two values smaller than 0.40 (G_{cut} = 0.16, 0.28, 0.69, 1.20).

As G_{cut} decreases two residues will be connected by an edge only if their correlation is very high. As a result the number of arcs of the network decreases. Consequently the number of paths connecting a given source node and a given target node is also expected to decrease. Consistently with this scenario when

G_{cut}=0.16 no path can be detected in any of the closed structures we produced. If G_{cut} is increased to 0.28 no path can be observed in 3 out of 4 subunits of the systems with gating charge 6e and 8e while in the fourth subunit we observe a path that is much longer and qualitatively different with respect to the path computed with G_{cut}=0.4. Interestingly, in the system with gating charge 4e the paths computed with G_{cut}=0.28 are qualitatively similar and of comparable length as compared to the paths computed with G_{cut}=0.4. It is thus likely that the edges lying along the minimal paths computed with G_{cut}=0.4 connected pairs of residues with such a highly correlated motion that they were not removed when G_{cut} was decreased from 0.40 to 0.28.

Conversely as G_{cut} increases, a smaller and smaller correlation is required for two residues to be linked by an edge in the graph. As a result, the number of edges of the network increases and also the number of paths connecting given end states is expected to grow. However, the minimal paths between a source and a target node tend to proceed along the shortest edges, i.e., those connecting the pairs of residues with the most correlated motion. As a result, minimal paths should to some extent be retained even in the face of an increase of G_{cut}, even if the presence of a greater number of edges allows shortcuts that decrease the length of the path. This is exactly what can be observed with G_{cut}=0.69 and G_{cut}=1.20: in most cases the minimal paths are qualitatively similar and their lengths are comparable or slightly shorter than those of the paths computed with G_{cut}=0.40. A few paths however, are qualitatively different from those computed with G_{cut}=0.40.

In hindsight, this analysis shows that the value G_{cut}=0.40 used in the article corresponds to the maximum value of correlation compatible with the establishment of efficient communication pathways. The paths computed with this value of G_{cut} are qualitatively retained up to G_{cut}=1.20 even if some alternative route starts to appear. The number of these alternative routes is expected to increase at even higher values of G_{cut}. The reliability of the results attained using a graph with edges connecting even weakly correlated pairs of residues, however, is highly questionable.

Supplementary Note 3: "A practical example of pathway calculation"

In order to clarify our protocol, in this section we provide a step-by-step example of pathway calculation. The data files used in this example

Reg1-Reg2_L45-Sub4_S6-Sub1.dat
Minpaths_L45-Sub4_S6-Sub1.dat
Centrality_L45-Sub4_S6-Sub1_sort.dat
Cont_Formation_Seq_filt_sort.dat

have been uploaded to the ZENODO repository where the PDB structure of the system with gating charge 4e where the computed paths can be mapped, can also be found.

The residue numbering appearing in this example is not the official one because the first Δ =397 residues of hERG (PAS domain) were not included in our simulations so that each subunit comprises L =466 residues. Moreover, the four subunits were numbered consecutively. The official numbering of a residue can be easily recovered. A residue $ires$ in our numbering is located on chain $ichain = ires/L + 1$ where we used the integer division. The correct numbering of this residue in its subunit is $ires_{true} = (iresL) + \Delta$ where indicates the modulus operation.

Suppose we want to compute some communication pathway in the system with gating charge Q_g =4e. We will have to do the following.

1. **Computation of the matrix of correlation coefficients**
   This matrix, C, is computed with a home-made program from the equilibrium simulation. The program also converts the map of correlation coefficients in the map of information distances $G_{ij} = -\log|C_{ij}|$ where

   $$\mathrm{C_{ij}} = \frac{\langle(\vec{r}_i - \langle\vec{r}_i\rangle)(\vec{r}_j - \langle\vec{r}_j\rangle)\rangle}{\sqrt{\langle(\vec{r}_i - \langle\vec{r}_i\rangle)^2\rangle\langle(\vec{r}_j - \langle\vec{r}_j\rangle)^2\rangle}} \qquad (1)$$

   with $\vec{r}_i$ and $\vec{r}_j$ being the position vectors of the i and j residues. The matrix G of information distances is stored in a file called mLog_Abs_Correl_Coeff.dat that is fed into another program that performs the proper path calculation.

2. **Determination of source and sink regions**
   The first task performed by the program for pathway calculation is the identification of the source and sink regions. To be more specific suppose we want to compute the communication pathways between loop L45 of subunit IV and helix S6 of subunit I. First of all, we need to choose a central residue in loop L45 and a central residue in helix S6. We chose residue 1541 in loop L45 and residue 261 in helix S6. The program then identifies all residues happening to spend at least 70% of the trajectory inside a sphere of radius 7.0 Å centered on these two residues. The residues, stored in a file called Reg1-Reg2_L45-Sub4_S6-Sub1.dat, are listed in Supplementary Table 3. The Table shows that residues 1539-1543 with consecutive numbering, are all located in the region we are interested in (loop L45) while residue 273 that is close to but outside loop L45, will be excluded by the analysis. In a similar way, residue 732 will be excluded by the sink region. In Supplementary Figure 22 the residues of the source and sink regions are mapped on the structure of the hERG channel (for the sake of graphical clarity only the S4-L45-S5 region of subunit IV and helix S6 of subunit I are shown).

3. **Calculation of minimal paths**
   Once the residues of the source and sink regions have been identified, the code applies Dijkstra's algorithm to compute a minimal length path for each pair i, j of residues with $i \in source$ and $j \in sink$. The most important inputs to the Dijkstra's subroutine are the matrix of information

distances G and a cutoff distance G_{cut}. In such a way the algorithm considers to be linked through an arc only those pairs of residues whose information distance G_{ij} is smaller than the chosen cutoff.

The procedure of network building from matrix G is illustrated in Supplementary Figure 23. In the trivial example of the figure G is a 4×4 matrix which means that the graph contains 4 nodes. Each row i of the matrix stores the information distances between node i and all other nodes j of the graph. In the example we assume a cutoff G_{cut} =5 so that only pairs of nodes with an information distance smaller than 5 will be connected through an arc. For example node 2 will be connected with node 1 (G_{21} =3< G_{cut} =5) and node 3 (G_{23} =2< G_{cut} =5) but not to node 4 (G_{24} =7> G_{cut} =5). The entries of the matrix act as the weights of the edges of the network. For instance the arc connecting nodes 1 and 3 has weight 4 because G_{13} =4.

The minimal paths thus, are computed using the matrix of information distances (that on turn derives from the matrix of correlation coefficients) and not the contact maps. The minimal paths are stored in a path named Minpaths_L45-Sub4_S6-Sub1.dat. Each line of this file has the following structure:

source, *targ*, N_{res}, *Length*, $res_1, res_2, \cdots, res_{N_{res}}$

where *source* and *targ* are the initial and final residues of the path, N_{res} is the number of residues traversed by the path, *length* is the path length (sum of the information distances of all the arcs of the path) and $res_1, res_2, \cdots, res_{N_{res}}$ is the sequence of residues actually placed along the path. For instance the second last line of the file

1543 265 5 0.8 1543 1546 1550 266 265

indicates a path from residue 1543 to residue 265; the path is composed by 5 residues and has a length 0.83. More specifically, the five residues traversed by the path are 1543, 1546, 1550, 266 and 265. This path, connecting the last residue of the source region with the last residue of the sink region is shown in Supplementary Figure 24. Even if all the paths belong to the same family and are qualitatively similar, choosing a representative path may not be easy. This task is simplified through the computation of the centrality index which is the next stage of the protocol.

4. **Calculation of Centrality Index**
When all the minimal paths have been computed, for each residue of the protein the code computes a centrality index *i.e.* the fraction of minimal paths the given residue belongs to. The residues with centrality index greater than zero are stored in file Centrality_L45-Sub4_S6-Sub1.dat. This file is sorted so that residues are ranked in order of increasing values of

the centrality index. If we arbitrarily choose only residues with centrality index greater than 0.15 and we ignore the residues found in paths starting from residue 273 (that we excluded from the source region) we find the following residues: 1539, 1540, 1541, 259, 261, 262, 1542, 1543, 266, 1546, 1550. As shown in Supplementary Figure 25 if these residues with the highest values of the Centrality Index are mapped on the structure of the channel, they form a pathway from loop L45 of subunit IV to helix S6 of subunit I. The Figure also shows a cartoon representation of the path with average length computed over all the minimal paths predicted by Dijkstra's algorithm.

5. **Pathway validation**

By now it is clear that the communication pathway was computed without the aid of contact maps. Indeed the graph used for the application of Dijkstra's algorithm was built using just two ingredients: the matrix of correlation coefficients and an appropriate cutoff, G_{cut} of the information distance. Once the path is computed, however, it is necessary to evaluate whether it represents a genuine avenue of propagation of a conformational transition. Indeed, since the calculation was completely based on the matrix of correlation coefficients, the residues lying along the computed path are characterized by a highly correlated motion. This however, is not sufficient for the path to be a route of propagation of a conformational transition. Actually, the residues lying next to each other along the path must also establish contacts so that the correlated motion can be expression of a causal influence on one another. For instance, from the top panel of Supplementary Figure 25 it appears that the uppermost residue of the pathway along helix S5 is rather distant from the lowermost residue of the path along helix S6. Thus, to make sure that there can be propagation of the motion from helix S5 to helix S6, it is necessary to analyze contacts between these two regions.

This is where the contact maps come into play. Indeed, it is rather difficult to spot a contact between a specific pair of residues watching a whole contact map. This is why the information of interest must be mined from the data file Cont_Formation_Seq_filt_sort.dat that provides the sequence of contact formation during the TMD trajectory from the open to the closed state. If we search this file for contacts involving helix S6 of subunit I we find, among others,

TYR 270 1 S6H VAL 1550 4 S5H 201

This line simply says that starting from frame 201 there is the stable establishment of a contact between Val1550 on helix S5 of subunit IV and Tyr270 on helix S1 of subunit I. This contact is critically important for the propagation of motion because both residues belong to the communication pathway and Val1550 is the residue with the highest value of the centrality

index, $C.I.$ =0.83. This means that Val1550 is involved in 83% of the minimal paths connecting loop L45 of subunit IV to helix S6 of subunit I. The visual inspection of the TMD trajectory further clarifies the role of this contact. The downward motion of helix S4 and the extension of loop L45, push up helix S5 of subunit IV reducing the distance between Val1550 and Tyr270. This enables the establishment of the Val1550-Tyr270 contact and through this contact, the propagation of motion.

Another interesting contact that emerges from the analysis of file Cont_Formation_Seq_filt_sort.dat is

THR 273 1 S7H SER 1544 4 L45 201

Even if Ser1544 does not sit on the communication pathway, it is the residue next to Tyr1543 which is the residue with the third highest centrality index, $C.I.$ =0.66. Thr273 on the other hand does not belong to helix S6 but to helix S7, so that this residue was not included in the sink region. Thr273, however is close to helix S6 and sink helix S7 is covalently connected to helix S6, this residue may play a role in the propagation of motion.

Supplementary Figures

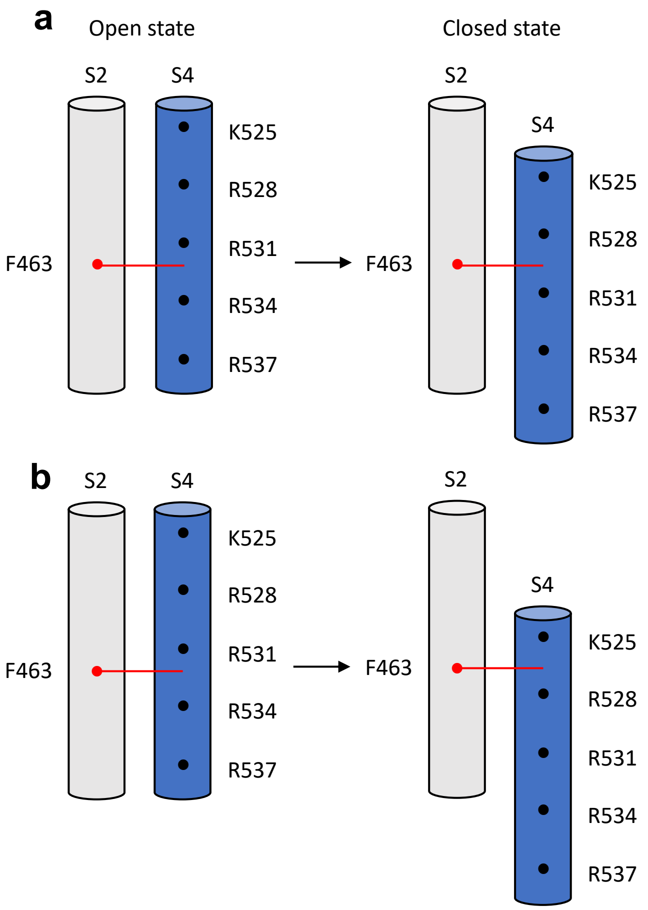

Supplementary Figure 1: Schematic representation of the displacement by one charged residue (**a**) and by two charged residues (**b**) to move the S4 helices in the closed configuration.

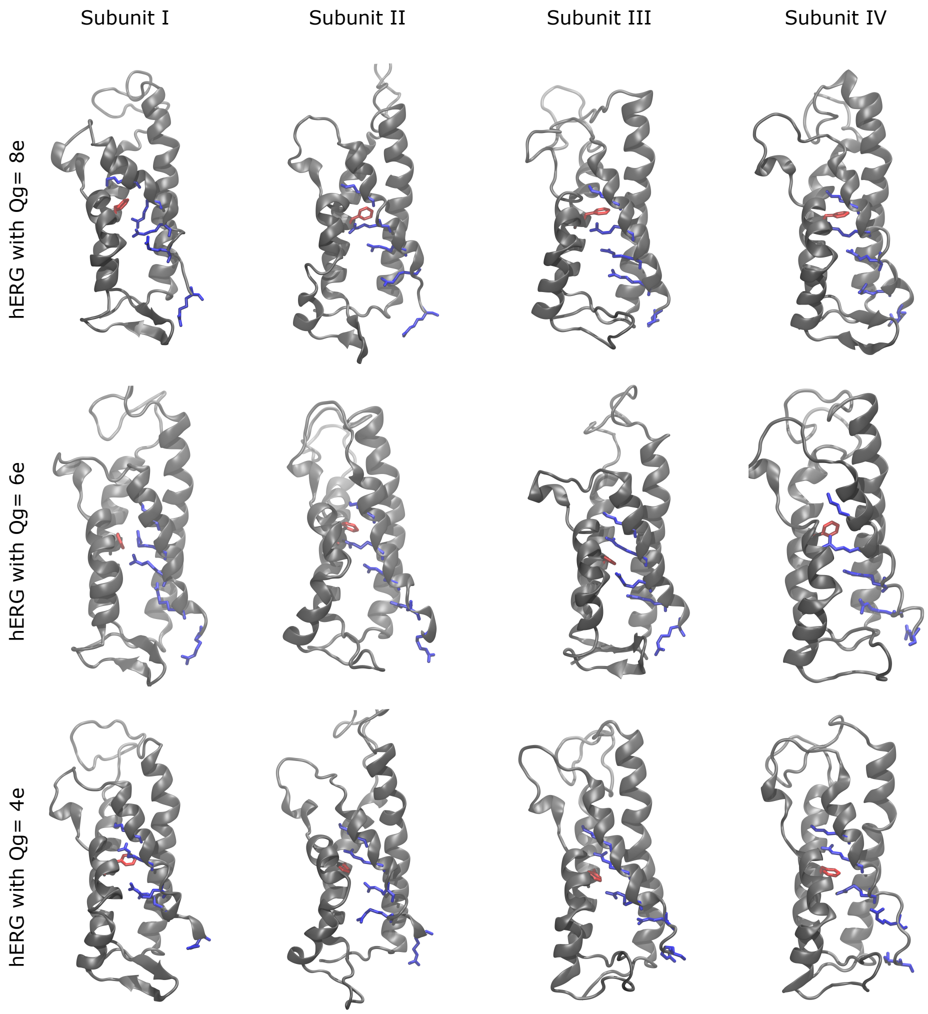

Supplementary Figure 2: S4 configuration in closed states after Steered MD simulations. The positive charged residues on helix S4 (K525, R528, R531, R534 and R537) are coloured in blue while the F463 on helix S2 that acts as the gating charge transfer centre is coloured in red.

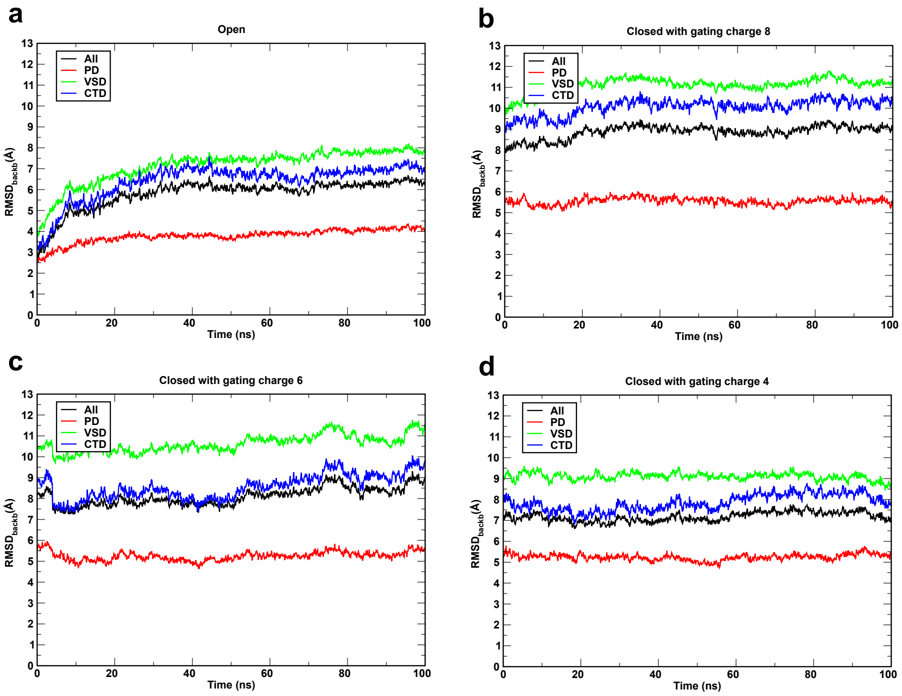

Supplementary Figure 3: The root-mean-square deviation (RMSD) during the 100 ns equilibrium simulations after the Steered MD simulations. Panels refer to: (**a**) open state; (**b**) closed state with $Q_g = 8$e; (**c**) closed state with $Q_g = 6$e; (**d**) closed state with $Q_g = 4$e. The RMSD plot of the open state was computed using as a reference conformation the experimental structure of hERG (PDB: 5VA2). For the RMSD profiles of closed states we used as reference structure, the homology model generated from the template EAG1 (PDB: 5K7L). The superposition of the current and reference conformations was performed using all backbone atoms, while the RMSD calculation was performed using different subsets of backbone atoms corresponding to different domains according to the following color code. Black line: all atoms; red line: pore domain atoms; green line: Voltage Sensor Domain atoms; blue line: Carboxy-Terminal Domain atoms.

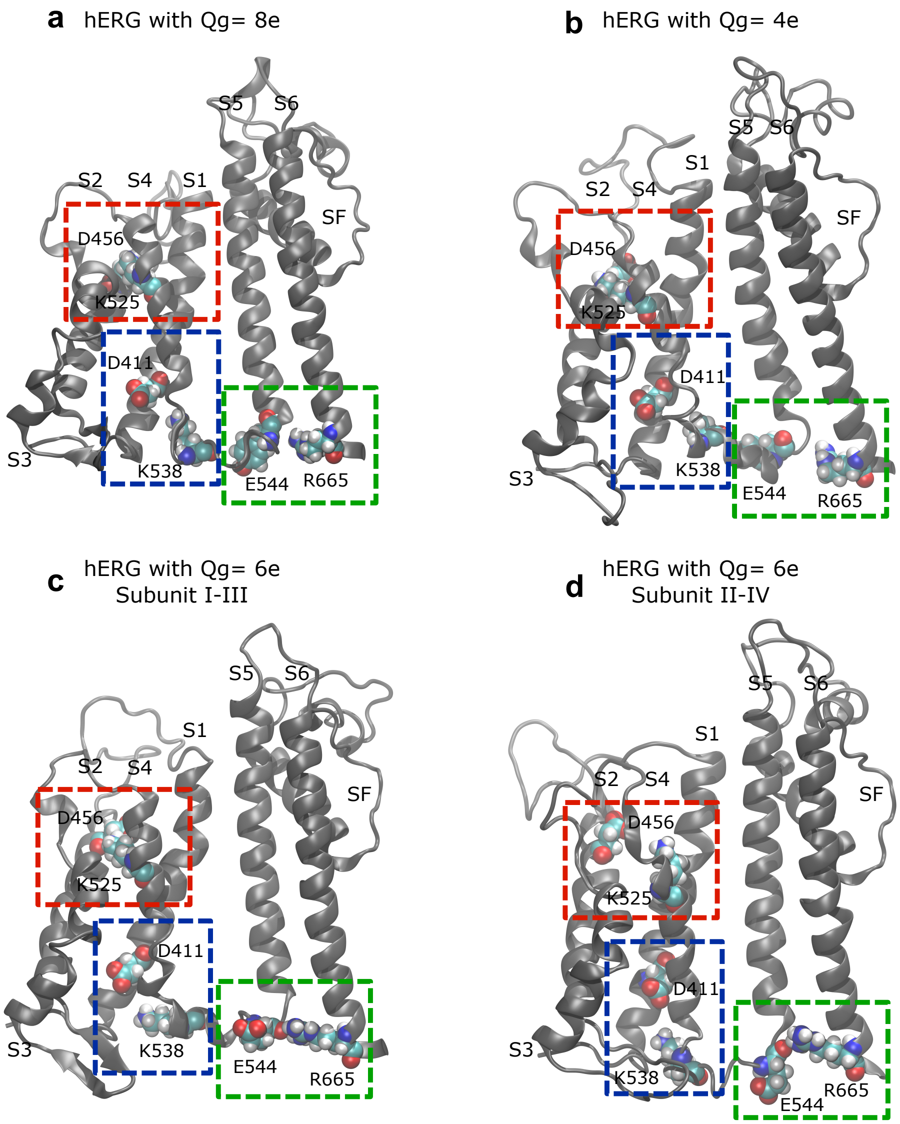

Supplementary Figure 4: Interactions known to stabilize the closed state of hERG: D456-K525 [1], D411-K538 [1] and E544-R665 [3, 6]. For simplicity, in systems with $Q_g = 8e$ (**a**) and $Q_g = 4e$ (**b**) the first subunit is shown after the equilibration; Panels **c** and **d** show the first and the second subunits of the system with $Q_g = 6e$ that are in the same configuration of the third and the fourth subunits respectively.

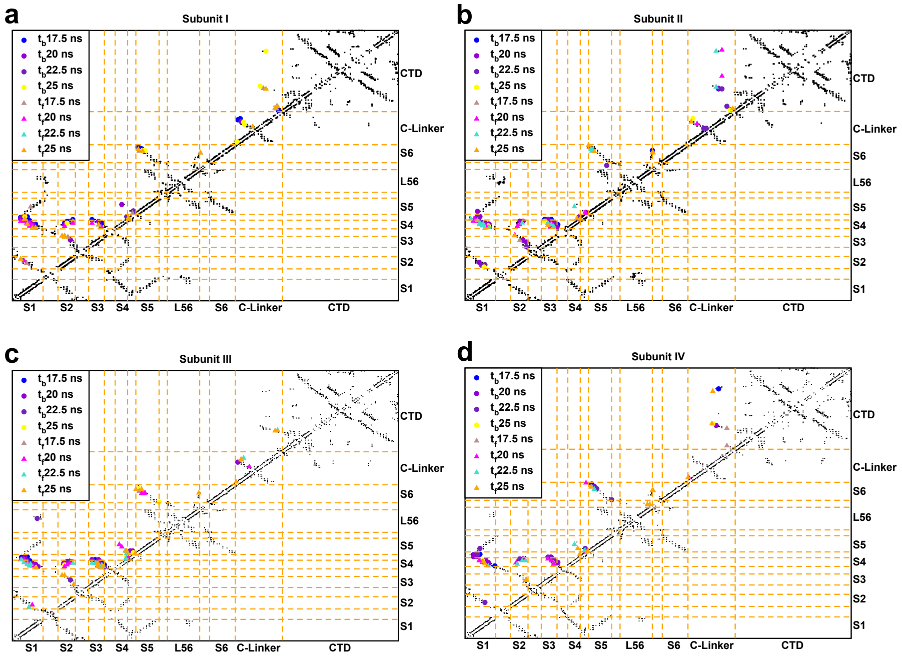

Supplementary Figure 5: Contact maps of each subunit of the system with $Q_g = 8e$ during the transition from O to C state. The black dots represent the contacts in the initial open conformation while the coloured symbols indicate the contacts formed or broken at different times t during the TMD simulation in the first subunit (**a**), in the second subunit (**b**), in the third subunit (**c**) and in the fourth subunit (**d**).

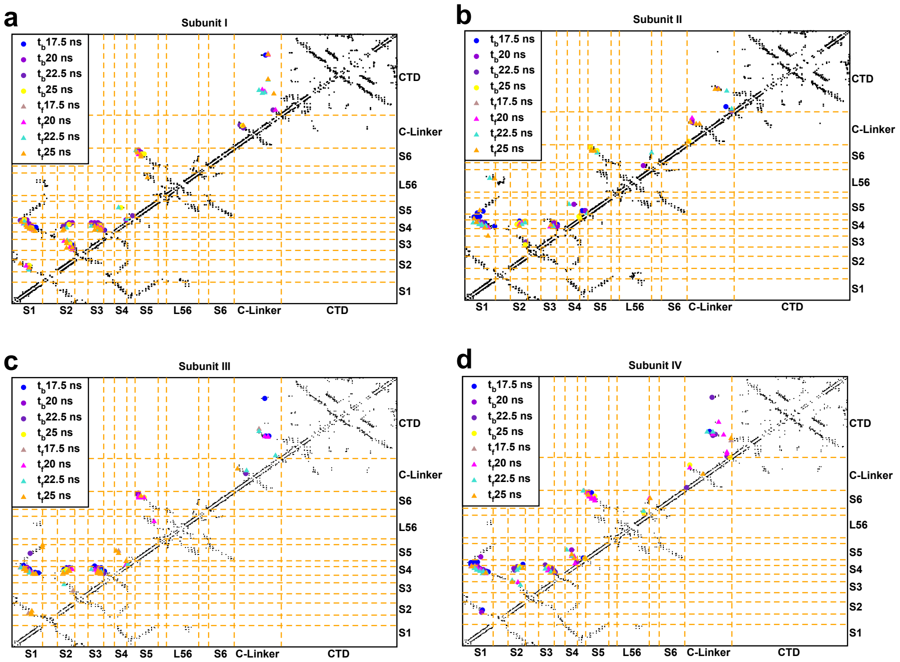

Supplementary Figure 6: Contact maps of each subunit of the system with $Q_g = 6e$ during the transition from O to C state. The black dots represent the contacts in the initial open conformation while the coloured symbols indicate the contacts formed or broken at different times t during the TMD simulation in the first subunit (**a**), in the second subunit (**b**), in the third subunit (**c**) and in the fourth subunit (**d**).

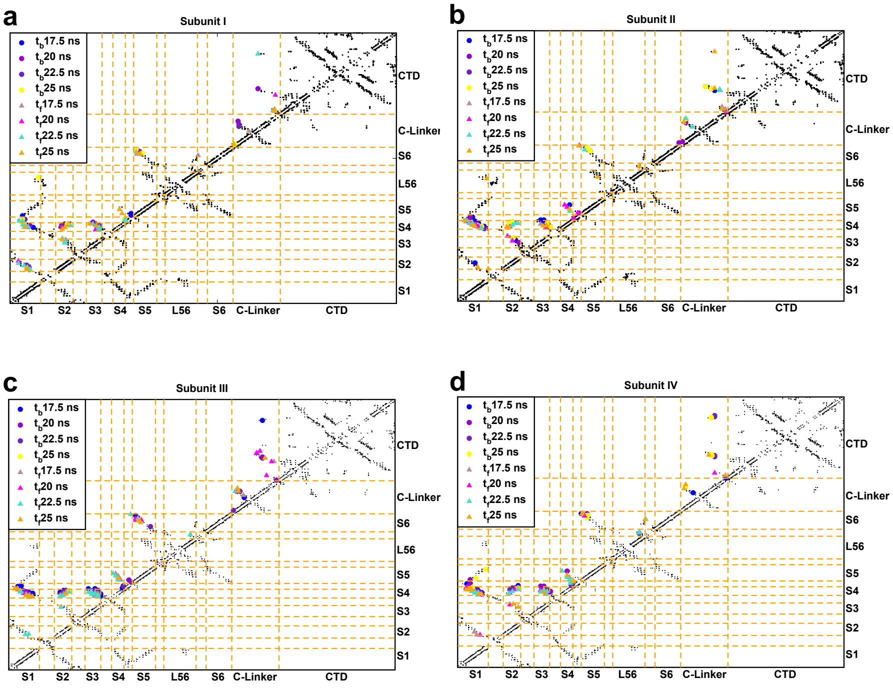

Supplementary Figure 7: Contact maps of each subunit of the system with $Q_g = 4e$ during the transition from O to C state. The black dots represent the contacts in the initial open conformation while the coloured symbols indicate the contacts formed or broken at different times t during the TMD simulation in the first subunit (**a**), in the second subunit (**b**), in the third subunit (**c**) and in the fourth subunit (**d**).

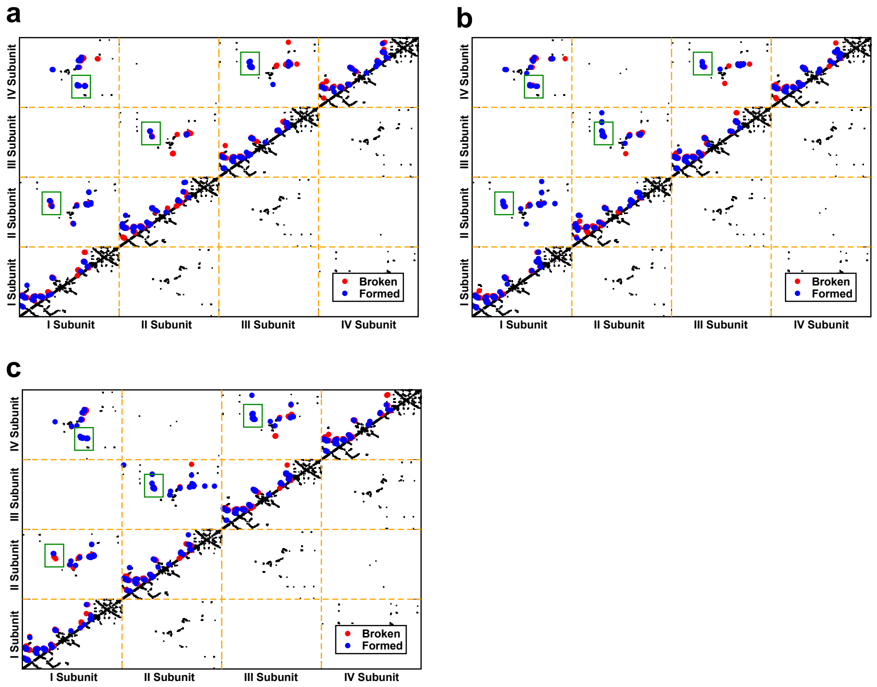

Supplementary Figure 8: Contact maps of the whole protein during the transition from O to C. Panels refer to systems with $Q_g = 8e$ (**a**), $6e$ (**b**), $4e$ (**c**). Conserved interactions between the loop L45 of subunit n and the C-Linker of subunit $n+1$ are highlighted in green boxes. The black dots represent the contacts in the initial open conformation while the coloured symbols indicate the contacts formed or broken.

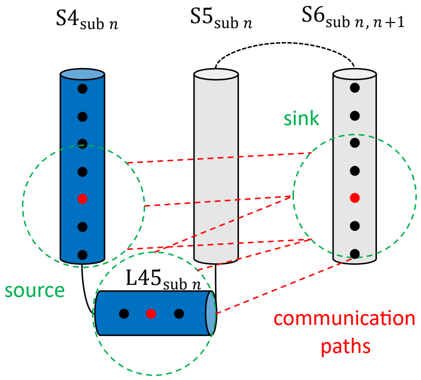

Supplementary Figure 9: Illustration of the communication path method; the source and sink regions are highlighted in green.

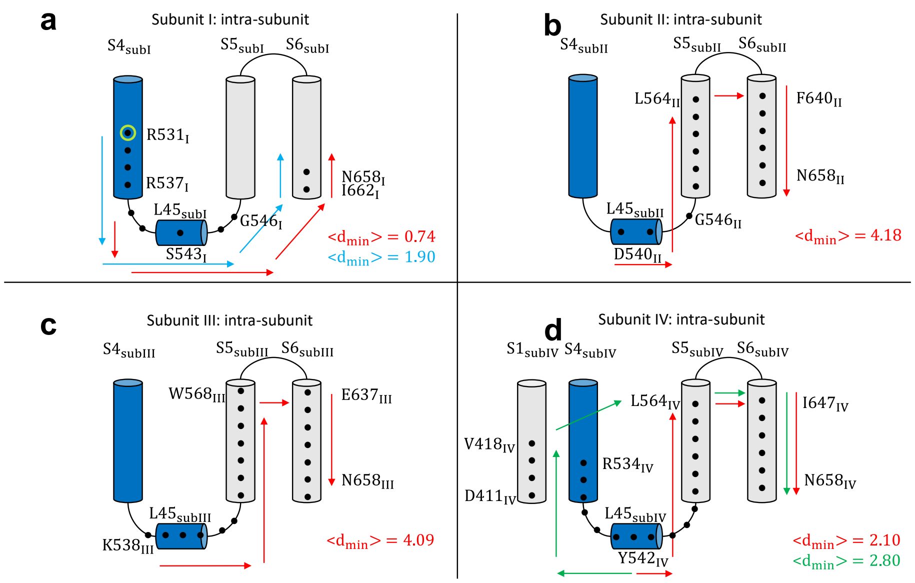

Supplementary Figure 10: Activation/deactivation pathways of the closed system with gating charge $6e$. Paths identified in the first subunit (**a**), in the second subunit (**b**), in the third subunit (**c**) and in the fourth subunit (**d**). Arrows describe the preferred routes of motion propagation: blue arrows refer to S4→L45→S6 route; red arrows refer to L45→S6 or L45→S5→S6 route; green arrows refer to S4→S1→S5→S6. Black dots correspond to residues on the path with $CI > 0.15$ (see Table S1); yellow circles refer to the pathological mutation R531Q/W [7] known to alter the gating of the channel inducing the LQTS. The mutations W410S, Y420C and T421M impair both the trafficking and the gating [8]. Average minimal path lengths $\langle d_{\min} \rangle$ are also reported.

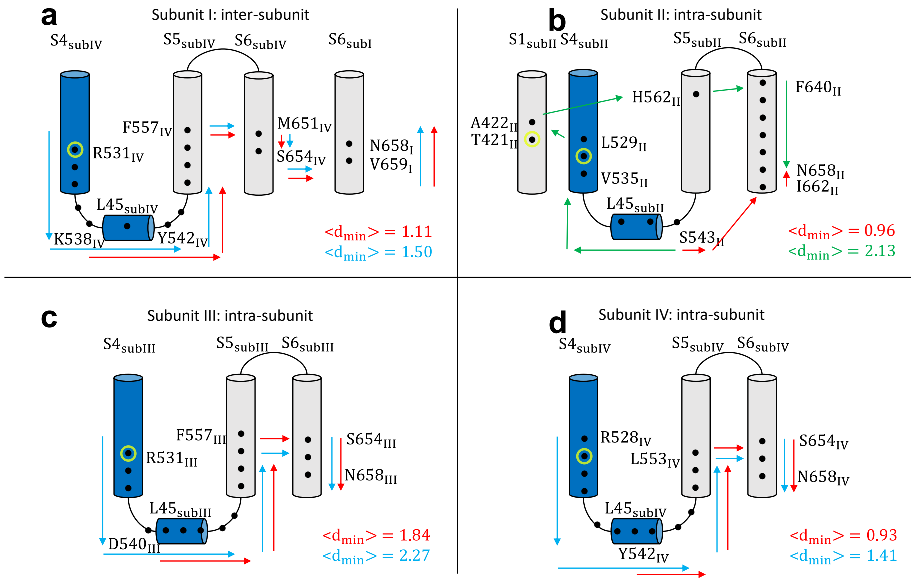

Supplementary Figure 11: Activation/deactivation pathways of the closed system with gating charge 4*e*. Paths identified in the first subunit (**a**), in the second subunit (**b**), in the third subunit (**c**) and in the fourth subunit (**d**). Arrows describe the preferred routes of motion propagation: blue arrows refer to S4→L45→S6 route; red arrows refer to L45→S6 or L45→S5→S6 route; green arrows refer to S4→S1→S5→S6. Black dots correspond to residues on the path with CI> 0.15 (see Table S1); yellow circles refer to the pathological mutation R531Q/W [7] known to alter the gating of the channel inducing the LQTS. The mutations W410S, Y420C and T421M impair both the trafficking and the gating [8]. Average minimal path lengths $\langle d_{\mathrm{min}} \rangle$ are also reported.

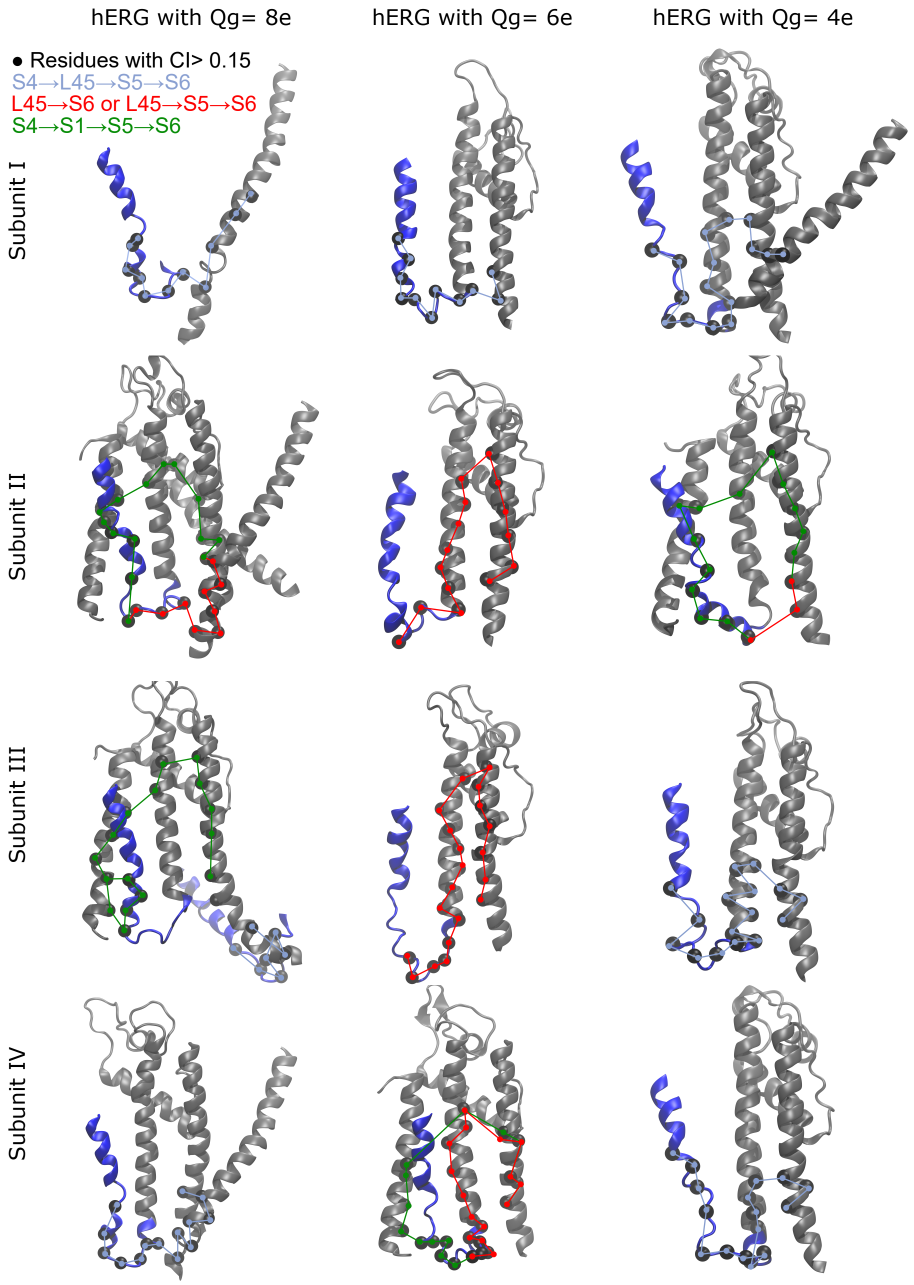

Supplementary Figure 12: Communication pathways of the motion propagation identified in hERG. S4 helix and loop L45 are coloured in blue; black dots are the protein residues on the path with CI> 0.15. The S4→L45→S5→S6 route is coloured in blue, the L45→S6 or L45→S5→S6 routes are in red and the S4→S1→S5→S6 path is in green.

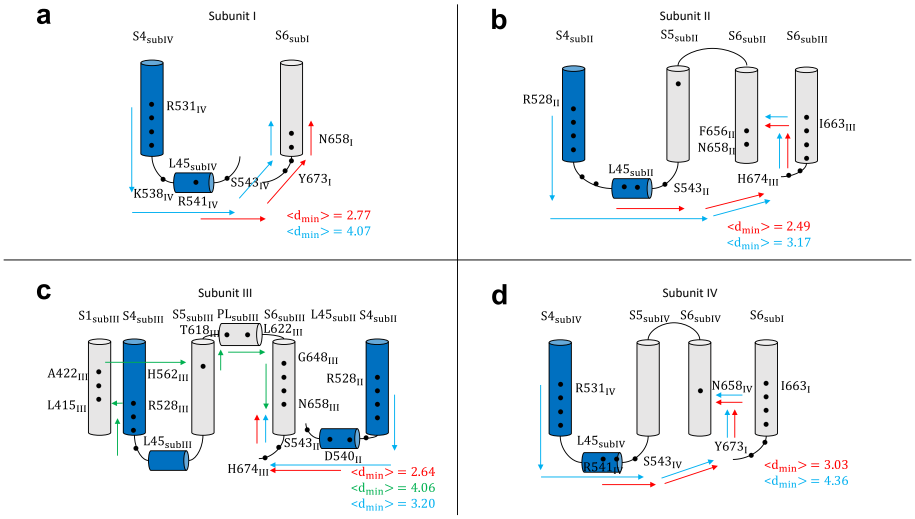

Supplementary Figure 13: Activation/deactivation pathways of the mutant T421M. Paths identified in the first subunit (**a**), in the second subunit (**b**), in the third subunit **(c)** and in the fourth subunit (**d**). Arrows describe the preferred routes of motion propagation: blue arrows refer to S4→L45→S5→S6 route; red arrows refer to L45→S6 or L45→S5→S6 route; green arrows refer to S4→S1→S5→S6. Black dots correspond to residues on the path with CI> 0.15. Average minimal path lengths $\langle d_{\min} \rangle$ are also reported.

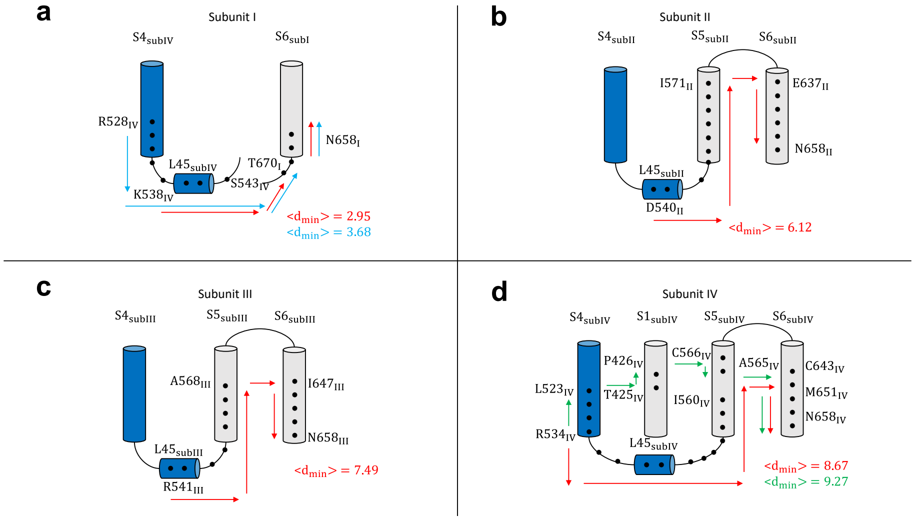

Supplementary Figure 14: Activation/deactivation pathways of the mutant R531Q. Paths identified in the first subunit (**a**), in the second subunit (**b**), in the third subunit **(c)** and in the fourth subunit (**d**). Arrows describe the preferred routes of motion propagation: blue arrows refer to S4→L45→S5→S6 route; red arrows refer to L45→S6 or L45→S5→S6 route; green arrows refer to S4→S1→S5→S6. Black dots correspond to residues on the path with CI> 0.15. Average minimal path lengths $\langle d_{\min} \rangle$ are also reported.

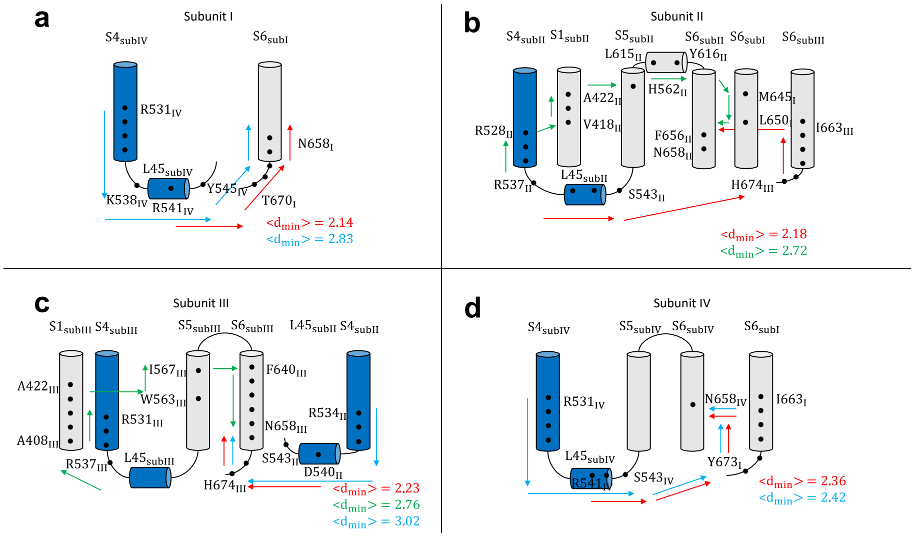

Supplementary Figure 15: Activation/deactivation pathways of the mutant A505V. Paths identified in the first subunit (**a**), in the second subunit (**b**), in the third subunit **(c)** and in the fourth subunit (**d**). Arrows describe the preferred routes of motion propagation: blue arrows refer to S4→L45→S5→S6 route; red arrows refer to L45→S6 or L45→S5→S6 route; green arrows refer to S4→S1→S5→S6. Black dots correspond-https://www.overleaf.com/project/60d61fb63faf1ddcf61427ee to residues on the path with CI> 0.15. Average minimal path lengths $\langle d_{\min} \rangle$ are also reported.

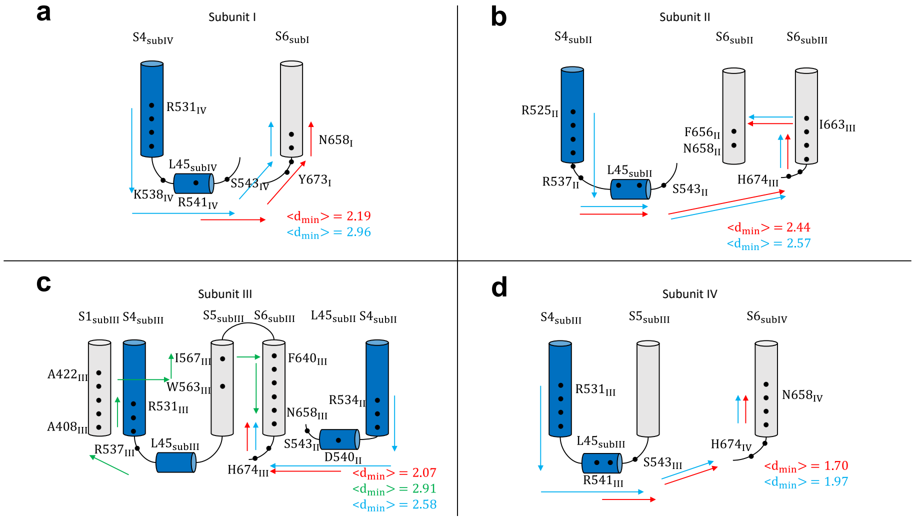

Supplementary Figure 16: Activation/deactivation pathways of the mutant F627A. Paths identified in the first subunit (**a**), in the second subunit (**b**), in the third subunit **(c)** and in the fourth subunit (**d**). Arrows describe the preferred routes of motion propagation: blue arrows refer to S4$\rightarrow$L45$\rightarrow$S5$\rightarrow$S6 route; red arrows refer to L45$\rightarrow$S6 or L45$\rightarrow$S5$\rightarrow$S6 route; green arrows refer to S4$\rightarrow$S1$\rightarrow$S5$\rightarrow$S6. Black dots correspond to residues on the path with CI$>$ 0.15. Average minimal path lengths $\langle d_{\min}\rangle$ are also reported.

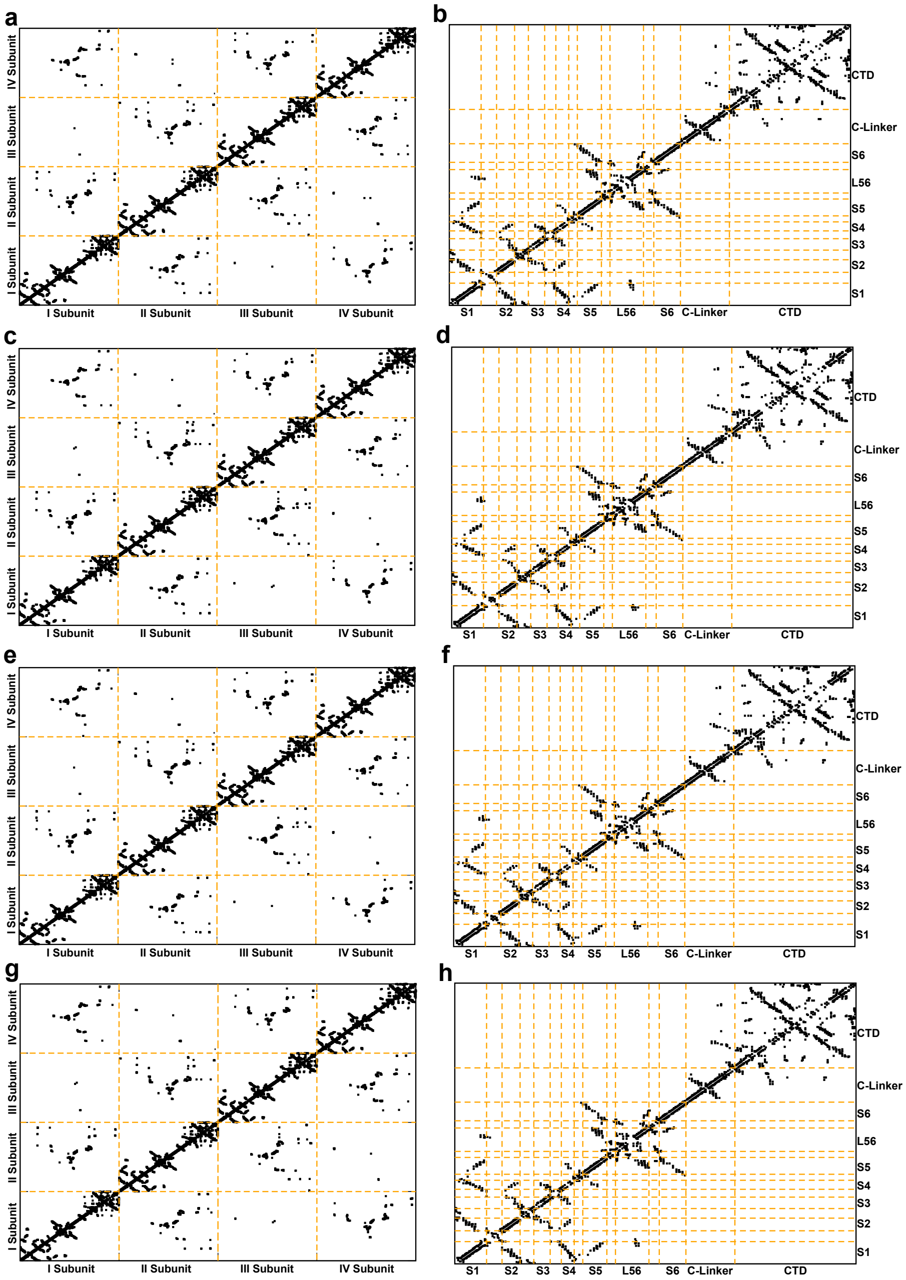

Supplementary Figure 17: Contact maps of the mutants T421M, R531Q, A505V and F627A. Panels **(a)** and **(b)** refer to T421M contact maps of the whole protein and of a single subunit, respectively. Panels **(c)** and **(d)** refer to R531Q contact maps of the whole protein and of a single subunit, respectively. Panels **(e)** and **(f)** refer to A505V contact maps of the whole protein and of a single subunit, respectively. Finally, panels **(g)** and **(h)** refer to F627A contact maps of the whole protein and of a single subunit, respectively. Black dots represent the formed interactions. In the single subunit maps the black dots are the formed interactions formed at least 75% of the trajectory in 3/4 subunits.

a

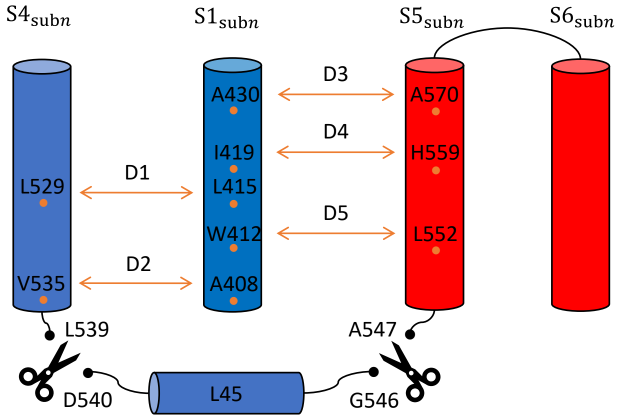

b

	D1	D2	D3	D4	D5
Open WT	~10 Å	~14 Å	~6 Å	~8 Å	~12 Å
Closed WT	~6 Å	~8 Å	~6 Å	~8 Å	~13 Å
Split D540	~6 Å	~14 Å	~8 Å	~10 Å	~15 Å
Split G546	~6 Å	~9 Å	~6 Å	~8 Å	~12 Å

Supplementary Figure 18: Distances between S4, S1 and S5 of the split channels. (**a**) Schematic representation of the computational systems highlighting the cutting points and residues as reference points for the distances between S4, S1 and S5; (**b**) Average distances between S4-S1 and S1-S5 in WT and split channels.

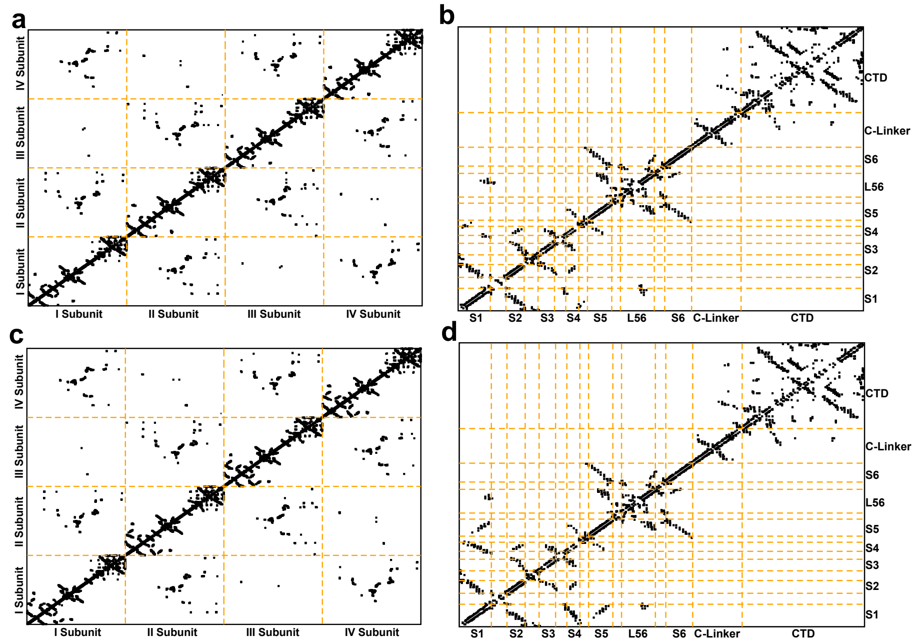

Supplementary Figure 19: Contact maps of the split channels. Panels **(a)** and **(b)** refer to the contact maps of the whole protein and of a single subunit in D540-cut channels; panels **(c)** and **(d)** refer to those in G546-cut channel. Black dots represent the formed interactions. In the single subunit maps the black dots are the formed interactions formed at least 75% of the trajectory in 3/4 subunits.

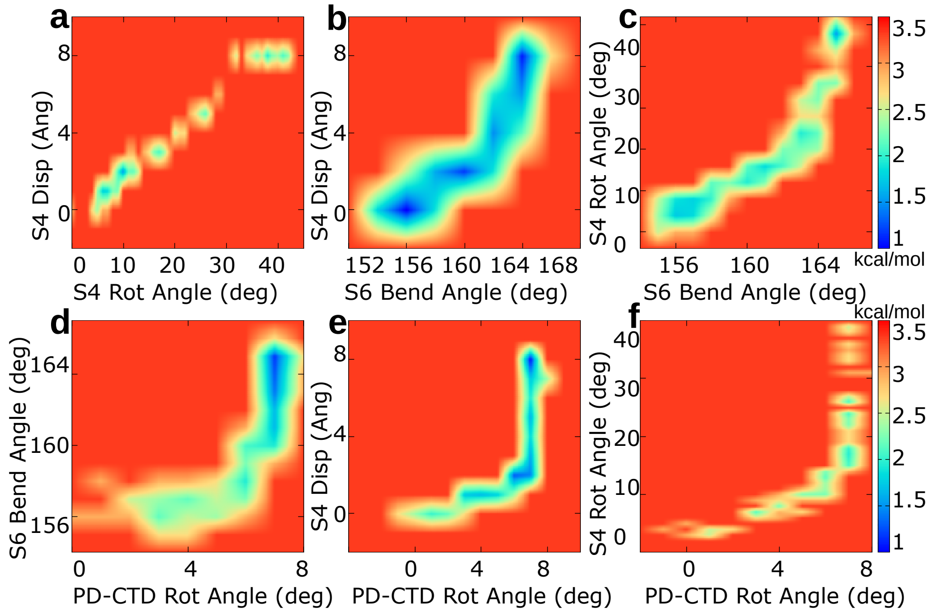

Supplementary Figure 20: Pseudo-potential of Mean Force for hERG with $Q_g = 6e$ as a function of geometrical descriptors, relevant for the O→C transition. Pseudo-PMF maps as follows: S4 rotation vs S4 displacement (**a**); S6 bending vs S4 displacement (**b**); S6 bending vs S4 rotation (**c**); PD-CTD rotation vs S6 bending (**d**); PD-CTD rotation vs S4 displacement (**e**); PD-CTD rotation vs S4 rotation (**f**). Dashed lines are a guide to the eye. Bin widths are as follows: S4 displacement: 1.0 Å; S4 rotation: 2°; S6 bending: 1°; PD-CTD rot: 1°. Free energies were expressed in kcal/mol.

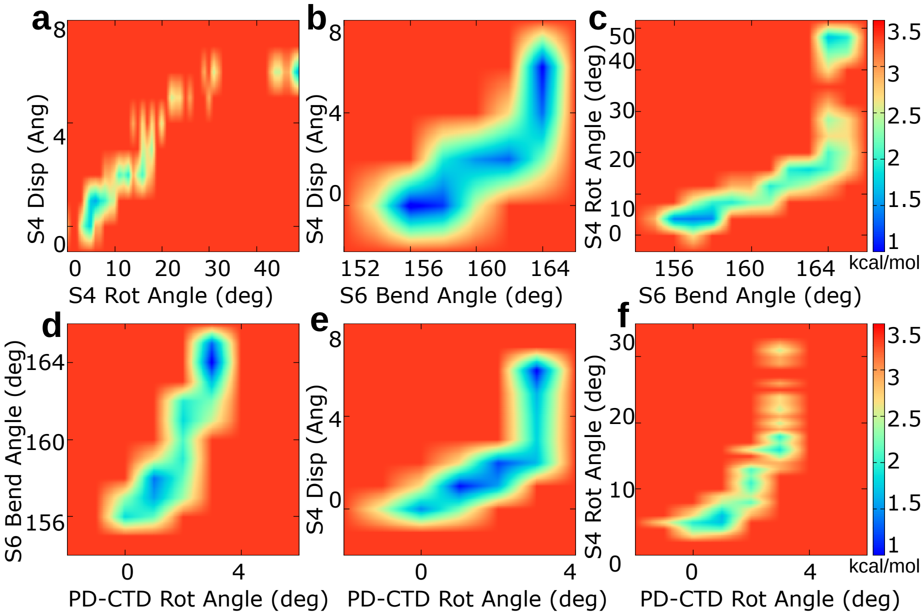

Supplementary Figure 21: Pseudo-potential of Mean Force for hERG with $Q_g = 4e$ as a function of geometrical descriptors, relevant for the O→C transition. Pseudo-PMF maps as follows: S4 rotation vs S4 displacement (**a**); S6 bending vs S4 displacement (**b**); S6 bending vs S4 rotation (**c**); PD-CTD rotation vs S6 bending (**d**); PD-CTD rotation vs S4 displacement (**e**); PD-CTD rotation vs S4 rotation (**f**). Dashed lines are a guide to the eye. Bin widths are as follows: S4 displacement: 1.0 Å; S4 rotation: 2°; S6 bending: 1°; PD-CTD rot: 1°. Free energies were expressed in kcal/mol.

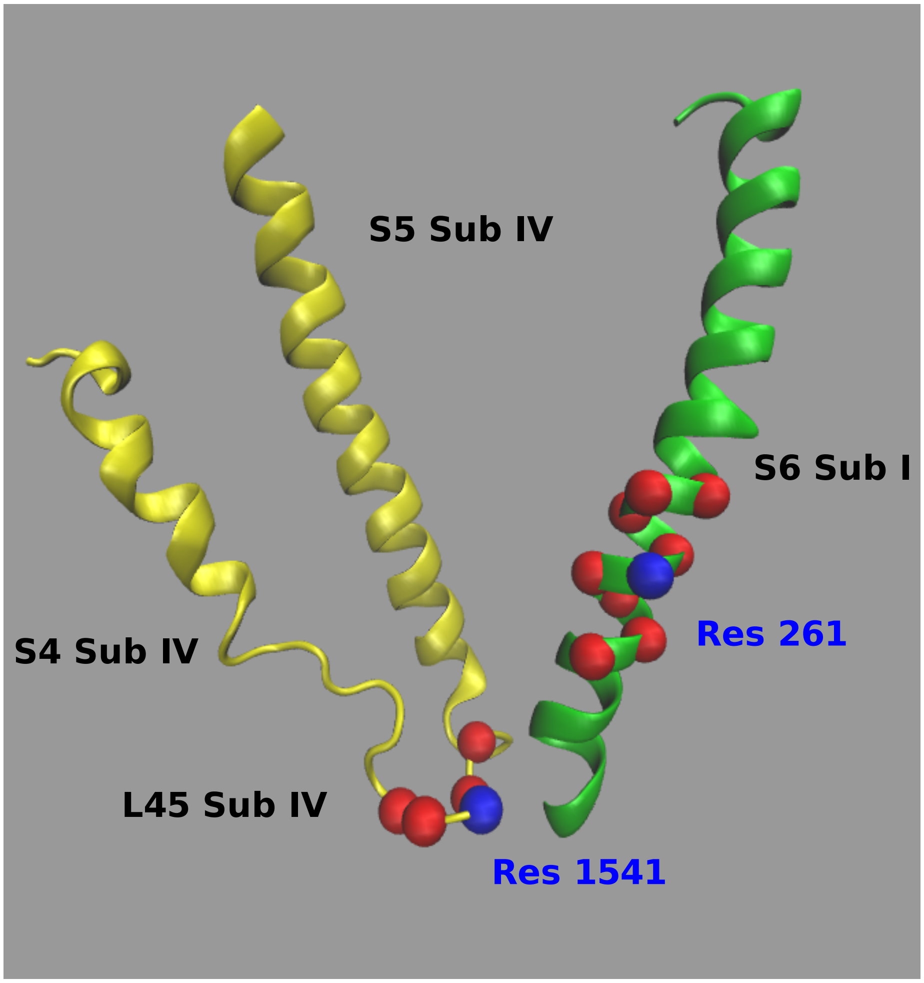

Supplementary Figure 22: Source and sink residues identified in loop L45 of subunit IV (yellow) and in helix S6 of subunit I (green). The central residues of the source and sink regions are shown as blue beads. The other residues of the source and sink regions (that can be found in at least 70% of the frames in a sphere of radius 7.0 Å centered on the central residues) are shown as red beads. See Supplementary Note 3 for more details.

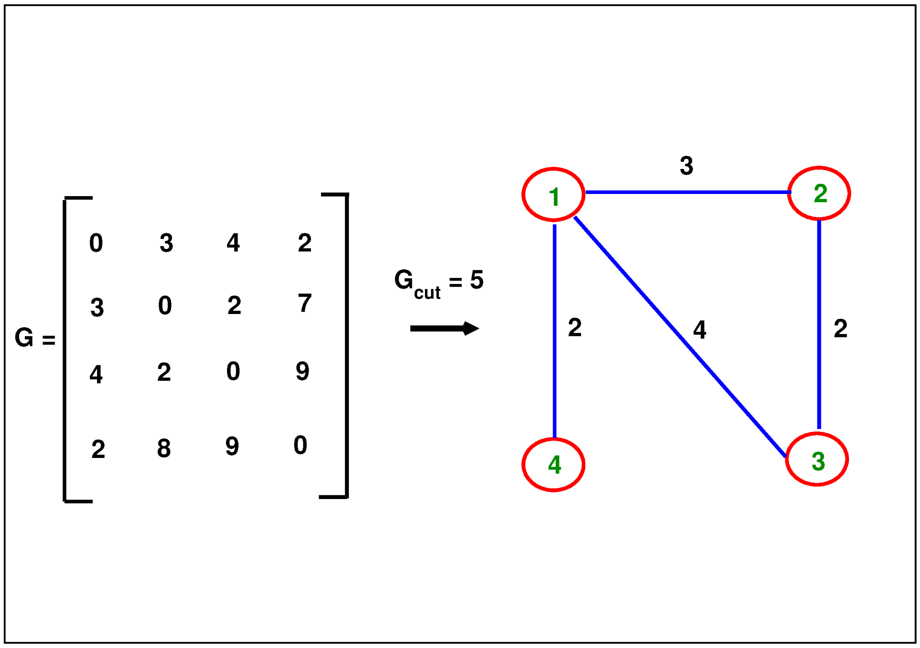

Supplementary Figure 23: Procedure of network building from the matrix G of information distances. Only pairs of nodes with an information distance $G_{ij} < G_{cut}$ will be connected through an arc. More details can be found in Supplementary Note 3.

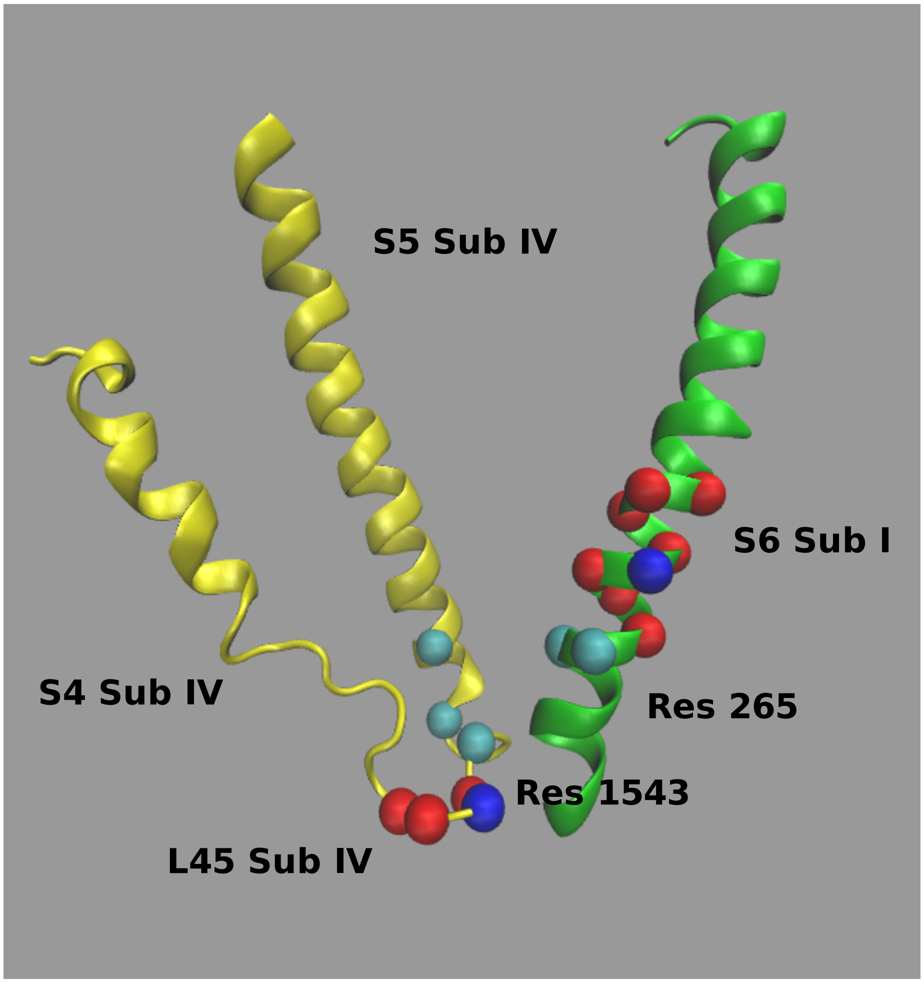

Supplementary Figure 24: An example of path (cyan beads) connecting residue 1543 in the source region with residue 265 in the sink region. The central residues of the source and sink region are shown in blue while the other residues of the two regions are coloured in red. For graphical clarity only the S4-L45-S5 region of subunit IV and helix S6 of subunit I are shown. For more detail see Supplementary Note 3.

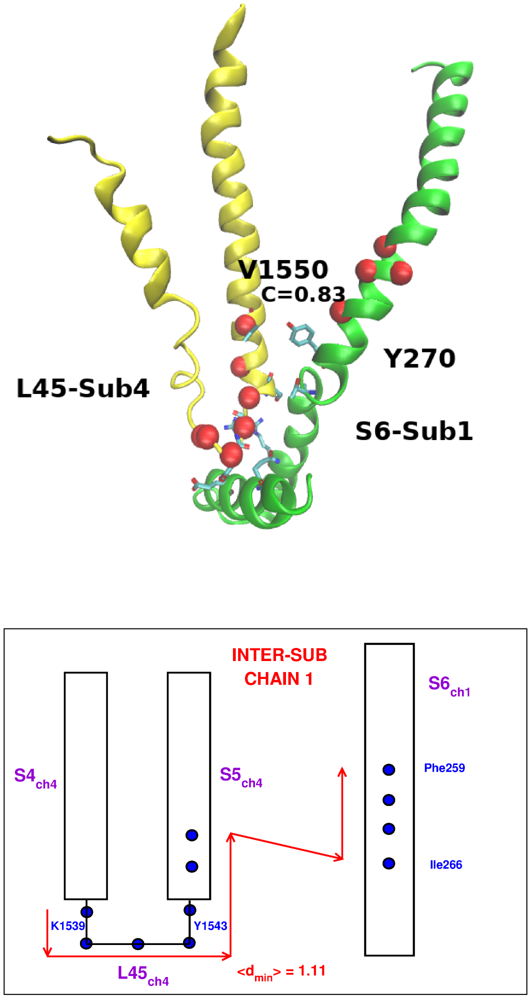

Supplementary Figure 25: Top panel: the distribution of residues with centrality index greater than 0.15 (red beads) outlines a communication pathway between loop L45 of subunit IV and helix S6 of subunit I. Residues involved in contacts between the two regions are portrayed in a stick representation. Lower panel: cartoon representation of the inter-subunit pathway ending on helix S6 of subunit I. The average path length was computed over all minimal paths computed with Dijkstra's algorithm. For more information see Supplementary Note 3.

		D456-K525	D411-K538	E544-R665
CLOSED $Q_g = 8$	Subunit I	6.16 Å	6.95 Å	3.99 Å
	Subunit II	4.91 Å	6.01 Å	3.61 Å
	Subunit III	4.82 Å	6.22 Å	4.26 Å
	Subunit IV	4.65 Å	5.38 Å	4.02 Å
CLOSED $Q_g = 6$	Subunit I	3.77 Å	4.48 Å	4.21 Å
	Subunit II	5.35 Å	6.89 Å	3.61 Å
	Subunit III	4.52 Å	4.69 Å	4.69 Å
	Subunit IV	4.77 Å	6.29 Å	4.57 Å
CLOSED $Q_g = 4$	Subunit I	5.23 Å	3.07 Å	3.27 Å
	Subunit II	4.38 Å	5.58 Å	3.65 Å
	Subunit III	6.09 Å	4.40 Å	5.34 Å
	Subunit IV	4.32 Å	5.84 Å	5.29 Å

Supplementary Table 1: Distances for the salt bridges in hERG closed systems. For D456-K525 and D411-K538 the values refer to the distance between the ϵ-Nitrogen of the lysines and the δ-Oxygen of the aspartates; for E544-R665 the values refer to the distance between the ϵ-Oxygen of the glutamates and the ϵ-Nitrogen of the arginines.

Residue	Centrality Index (CI)	Betweenness
A408	$CI \geq 0.95$	High
W410	$CI \geq 0.95$	High
D411	$CI \geq 0.95$	High
V418	$CI \geq 0.95$	High
Y420	$CI \geq 0.95$	High
T421	$CI \geq 0.95$	High
A422	$CI \geq 0.95$	High
R528	$0.85 \leq CI \leq 0.95$	High
L529	$0.85 \leq CI \leq 0.95$	High
R531	$0.55 \leq CI \leq 0.65$	Medium
R534	$0.35 \leq CI \leq 0.45$	Medium
V535	$0.25 \leq CI \leq 0.45$	Medium
R537	$CI \geq 0.95$	High
K538	$CI \geq 0.95$	High
D540	$CI \geq 0.95$	High
R541	$CI \geq 0.95$	High
Y542	$CI \geq 0.95$	High
S543	$CI \geq 0.95$	High
E544	$CI \geq 0.95$	High
G546	$CI \geq 0.95$	High
L552	$CI \geq 0.95$	High
L553	$0.85 \leq CI \leq 0.95$	Medium
F557	$0.85 \leq CI \leq 0.95$	Medium
A558	$0.70 \leq CI \leq 0.80$	Medium
L559	$0.70 \leq CI \leq 0.80$	Medium
H562	$CI \geq 0.95$	High
W563	$0.80 \leq CI \leq 0.90$	High
L564	$0.80 \leq CI \leq 0.90$	Medium
I567	$0.80 \leq CI \leq 0.90$	Medium
W568	$CI \geq 0.95$	High
L615	$CI \geq 0.95$	Medium
Y616	$CI \geq 0.95$	Medium
E637	$0.75 \leq CI \leq 0.85$	Medium
F640	$0.70 \leq CI \leq 0.80$	Medium
V644	$0.70 \leq CI \leq 0.80$	Medium
M645	$CI \geq 0.95$	High
I647	$CI \geq 0.95$	High
L650	$CI \geq 0.95$	High
M651	$CI \geq 0.95$	High
S654	$0.45 \leq CI \leq 0.55$	Medium
F656	$0.75 \leq CI \leq 0.85$	Medium
N658	$CI \geq 0.95$	High
I662	$CI \geq 0.95$	High
I663	$CI \geq 0.95$	High
Y673	$CI \geq 0.95$	High
H674	$CI \geq 0.95$	High

Supplementary Table 2: Centrality Index (CI) and betweenness (B) of each residue implicated in the paths. Legend betweenness (B): low $0 < B \leq 1$; medium: $1 < B \leq 4$; high: $4 < B \leq B_{max}$

Source residues	
Serial index	Residue number
1	273
2	1539
3	1540
4	1541
5	1542
6	1543
Sink residues	
1	257
2	258
3	259
4	260
5	261
6	262
7	263
8	264
9	265
10	732

Supplementary Table 3: Source and sink residues identified for the calculation of communication pathways between loop L45 of Subunit IV and helix S6 of Subunit I in the system with gating charge 4e. See Supplementary Note 3 for more details.

	$Q_g = 4e$	$Q_g = 6e$	$Q_g = 8e$
Min Variance in Whole Matrix	0.000	0.000	0.000
Max Variance in Whole Matrix	20.518	12.569	16.311
Avg Variance in Whole Matrix	0.296	0.292	0.293
Min Variance in the Network	6.657 x 10-6	1.504 x 10-5	3.404 x 10-6
Max Variance in the Network	20.518	12.569	16.311
Avg Variance in the Network	0.301	0.297	0.297

Supplementary Table 4: Minimum, maximum and average value of the variance both considering all elements of the matrix or just the most correlated pairs of residues ($< (\bar{G_{ij}})_B >< G_{cut}$).

System with $Q_g = 8e$		
Residues on the Inter-Subunit path S4(IV)→L45(IV)→S6(I)	Average	Variance of the Mean
R531-R534	0.5503	5.2522 x 10-3
R534-V535	0.1603	1.0749 x 10-3
V535-R537	0.4014	1.6772 x 10-3
R537-K538	0.1578	1.7875 x 10-3
K538-R541	0.4271	1.0073 x 10-3
R541-S543	0.3201	1.7564 x 10-3
S543-Y673	0.3478	5.1914 x 10-2
Y673-I663	0.3180	7.0954 x 10-2
I663-I662	0.2040	2.0358 x 10-3
I662-N658	0.3289	1.1647 x 10-3
Residues on the Intra-Subunit path S4(III)→S1(III)→S5(III)→S6(III)	Average	Variance of the Mean
R537-V535	0.2719	1.7215 x 10-1
V535-R534	0.1443	2.3312 x 10-3
R534-R531	0.2689	1.1762 x 10-3
R531-A408	0.5191	1.9440 x 10-2
A408-W410	0.2125	1.6261 x 10-3
W410-D411	0.1252	1.6004 x 10-3
D411-T421	0.6234	1.0774 x 10-2
T421-A422	0.1854	3.3200 x 10-3
A422-W563	0.3974	7.5543 x 10-4
W563-I567	0.2156	5.2802 x 10-4
I567-F640	0.4552	2.7933 x 10-3
F640-V644	0.3478	4.3948 x 10-3
V644-I647	0.2950	7.3684 x 10-4
I647-F656	0.5306	1.0186 x 10-2
F656-N658	0.3529	2.8043 x 10-3

Supplementary Table 5: Variance analysis of the pathways for the systems with gating charge $Q_g = 8e$.

System with $Q_g = 6e$		
Residues on the Intra-Subunit path S4(I)→L45(I)→S6(I)	Average	Variance of the Mean
R531-R534	0.5653	2.2978 x 10-3
R534-V535	0.1875	2.6695 x 10-4
V535-R537	0.5656	2.3842 x 10-2
R537-K538	0.2387	1.0461 x 10-3
K538-Y542	0.9294	0.1269
Y542-S543	0.2178	2.3915 x 10-3
S543-E544	0.2124	1.7447 x 10-3
E544-G546	0.4526	5.6113 x 10-3
G546-I662	0.7260	2.5726 x 10-2
I662-N658	0.2858	4.6942 x 10-3
Residues on the Intra-Subunit path S4(IV)→S1(IV)→S5(IV)→S6(IV)	Average	Variance of the Mean
Y542-R541	0.3779	1.5757 x 10-2
R541-D540	0.1257	2.2496 x 10-5
D540-K538	0.4351	2.0983 x 10-2
K538-R537	0.2229	3.4329 x 10-3
R537-V535	0.4212	3.6303 x 10-2
V535-R534	0.2488	9.4591 x 10-3
R534-D411	0.5707	7.1781 x 10-2
D411-I414	0.1680	1.4495 x 10-3
I414-L416	0.1599	7.1927 x 10-4
L416-V418	0.1984	1.4448 x 10-3
V418-L564	0.6238	4.2837 x 10-3
L564-I647	0.7336	4.7687 x 10-3
I647-L650	0.6742	1.0124 x 10-2
L650-M651	0.2968	1.0005 x 10-3
M651-S654	0.3196	1.4592 x 10-3
S654-F656	0.3064	1.1015 x 10-3
F656-N658	0.2469	1.5892 x 10-3

Supplementary Table 6: Variance analysis of the pathways for the systems with gating charge $Q_g = 6e$.

System with $Q_g = 4e$		
Residues on the Intra-Subunit path S4(II)→S1(II)→S5(II)→S6(II)	Average	Variance of the Mean
S543-D540	0.8007	5.7774 x 10-2
D540-K538	0.3639	2.7237 x 10-3
K538-V535	0.4844	1.0767 x 10-2
V535-R531	0.4509	9.1011 x 10-3
R531-L529	0.2687	2.8274 x 10-3
L529-T421	0.4868	3.2800 x 10-3
T421-A422	0.1364	1.0259 x 10-4
A422-H562	0.4096	1.4083 x 10-3
H562-F640	0.7829	4.9195 x 10-3
F640-V644	0.3511	1.7799 x 10-3
V644-I647	0.3106	2.0210 x 10-3
I647-M651	0.4589	1.8192 x 10-3
M651-S654	0.3247	2.2810 x 10-3
S654-N658	0.2982	6.3588 x 10-4
Residues on the Intra-Subunit path S4(III)→L45(III)→S5(III)→S6(III)	Average	Variance of the Mean
R531-R534	0.5804	2.2022 x 10-2
R534-V535	0.2176	4.2242 x 10-4
V535-K538	0.8161	4.5097 x 10-3
K538-D540	0.4147	5.0377 x 10-3
D540-R541	0.1861	2.4843 x 10-5
R541-Y542	0.1783	1.1731 x 10-3
Y542-S543	0.2066	7.0971 x 10-4
S543-E544	0.1962	1.7119 x 10-4
E544-G546	0.4971	1.3107 x 10-2
G546-L552	0.4719	1.7032 x 10-2
L552-L553	0.1181	2.5540 x 10-4
L553-F557	0.2328	1.2514 x 10-3
F557-S654	0.7629	3.9073 x 10-2
S654-F656	0.4206	7.2767 x 10-3
F656-N658	0.2547	8.0104 x 10-4

Supplementary Table 7: Variance analysis of the pathways for the systems with gating charge $Q_g = 4e$.

Supplementary References

[1] M. Zhang, J. Liu, M. Jiang, D.-M. Wu, K. Sonawane, H. Guy, and G.-N. Tseng, "Interactions between charged residues in the transmembrane segments of the voltage-sensing domain in the hERG channel," *The Journal of membrane biology*, vol. 207, no. 3, pp. 169–181, 2005.

[2] D. R. Piper, A. Varghese, M. C. Sanguinetti, and M. Tristani-Firouzi, "Gating currents associated with intramembrane charge displacement in HERG potassium channels," *Proceedings of the National Academy of Sciences*, vol. 100, no. 18, pp. 10534–10539, 2003.

[3] M. Sanguinetti and Q. Xu, "Mutations of the S4-S5 linker alter activation properties of HERG potassium channels expressed in Xenopus oocytes," *The Journal of Physiology*, vol. 514, no. 3, pp. 667–675, 1999.

[4] M. Tristani-Firouzi, J. Chen, and M. C. Sanguinetti, "Interactions between S4-S5 linker and S6 transmembrane domain modulate gating of HERG K+ channels," *Journal of Biological Chemistry*, vol. 277, no. 21, pp. 18994–19000, 2002.

[5] J. Guo, S. Durdagi, M. Changalov, L. L. Perissinotti, J. M. Hargreaves, T. G. Back, S. Y. Noskov, and H. J. Duff, "Structure driven design of novel human ether-a-go-go-related-gene channel (hERG1) activators," *PloS one*, vol. 9, no. 9, p. e105553, 2014.

[6] O. A. Malak, Z. Es-Salah-Lamoureux, and G. Loussouarn, "hERG S4-S5 linker acts as a voltage-dependent ligand that binds to the activation gate and locks it in a closed state," *Scientific reports*, vol. 7, no. 1, pp. 1–12, 2017.

[7] C. M. McBride, A. M. Smith, J. L. Smith, A. R. Reloj, E. J. Velasco, J. Powell, C. S. Elayi, D. C. Bartos, D. E. Burgess, and B. P. Delisle, "Mechanistic basis for type 2 long QT syndrome caused by KCNH2 mutations that disrupt conserved arginine residues in the voltage sensor," *The Journal of membrane biology*, vol. 246, no. 5, pp. 355–364, 2013.

[8] K. Phan, C. A. Ng, E. David, D. Shishmarev, P. W. Kuchel, J. I. Vandenberg, and M. D. Perry, "The S1 helix critically regulates the finely tuned gating of Kv11. 1 channels," *Journal of Biological Chemistry*, vol. 292, no. 18, pp. 7688–7705, 2017.
