# Supplementary material for: Molecular dynamics simulations suggest possible activation and deactivation pathways in the hERG channel
Source: Commun Biol. 2022 Feb 24;5:165. doi: 10.1038/s42003-022-03074-9 (PMC8873449; doi:10.1038/s42003-022-03074-9)
Supplement: Supplementary file 2 — Description of Additional Supplementary Files [file 42003_2022_3074_MOESM2_ESM.pdf]

Description of Additional Supplementary Files

File name: Supplementary Movie 1
Description: Inter-subunit path of the system with Qg = 8e described by blue arrows in Figure 3a: the motion propagates downwards along helix S4 and then along loop L45 of the fourth subunit. From there, it jumps directly to helix S6 of the first subunit. Residues highlighted have a CI> 0.15.

File name: Supplementary Movie 2
Description: Intra-subunit path of the system with Qg = 8e described by green arrows in Figure 3c: the motion propagates upwards along helix S4 of the third subunit before moving to S1 and S5 of the same subunit. From there, the path reached the helix S6 of the same subunit. Residues highlighted have a CI> 0.15.

File name: Supplementary Movie 3
Description: Intra-subunit path of the system with Qg = 4e described by green arrows in Supplementary Figure 11b: the motion propagates upwards along helix S4 of the second subunit before moving to S1 and S5 of the same subunit. From there, the path reached the helix S6 of the same subunit. Residues highlighted have a CI> 0.15.

File name: Supplementary Movie 4
Description: Intra-subunit path of the system with Qg = 4e described by blue arrows in Supplementary Figure 11c: the motion propagates downwards along helix S4 and then along loop L45 of the third subunit. From there, it goes on helix S5 to jump to helix S6 of the same subunit. Residues highlighted have a CI> 0.15.
